# GAPDH nuclear signaling regulates neutrophil migration and inflammatory skin responses

**DOI:** 10.21203/rs.3.rs-6170527/v1

**Published:** 2026-08-11

**Authors:** Joaquín Cantón-Sandoval, Francisco J. Martínez-Morcillo, Natalia Pérez-Escudero, Isabel Cabas, Beatriz Bernal-Bermúdez, Anna Lucianò, Juan M. Lozano-Gil, Alicia Martínez-López, Cassia Michael, Teresa Martínez-Menchón, Raúl Corbalán-Vélez, Belén Ferri, María L. Cayuela, Raquel Espín-Palazón, Sofia de Oliveira, Diana García-Moreno, Victoriano Mulero

**Affiliations:** 1. Inmunidad, Inflamación y Cáncer. Departamento de Biología Celular e Histología, Facultad de Biología, Universidad de Murcia. 30100 Murcia, Spain.; 2. Instituto Murciano de Investigación Biosanitaria (IMIB)-Pascual Parrilla, 30120 Murcia, Spain.; 3. Centro de Investigación Biomédica en Red de Enfermedades Raras (CIBERER), Instituto de Salud Carlos III, 28029 Madrid, Spain.; 4. CEIR Campus Mare Nostrum (CMN), Universidad de Murcia.; 5. Department of Developmental and Molecular Biology, Albert Einstein College of Medicine; Department of Medicine (Hepatology), Albert Einstein College of Medicine. New York. USA.; 6. Hospital Clínico Universitario Virgen de la Arrixaca. 30120 Murcia, Spain.; 7. Department of Genetics, Development and Cell Biology; Iowa State University, Ames, IA, 50011, USA.

**Keywords:** skin inflammation, neutrophils, GAPDH, oxidative stress, interleukin-4, psoriasis, atopic dermatitis

## Abstract

Neutrophils are the most abundant leukocytes and key regulators of tissue homeostasis and disease. Here we show that inhibiting Gapdh nuclear translocation limits neutrophil infiltration into inflamed skin, reduces their motility, and decreases keratinocyte hyperproliferation and cell death in a zebrafish model of skin inflammation. Wild-type Gapdh expression in neutrophils, but not a nuclear-translocation–deficient mutant, exacerbated inflammation. Pharmacological inhibition of GAPDH nuclear translocation also attenuated pathology-associated markers in human organotypic 3D skin models of inflammation. In psoriasis lesions, GAPDH levels were elevated with altered localization. Single-cell RNA-seq revealed that Gapdh nuclear translocation regulates a subset of inflammation-induced genes in neutrophils, including *il4*. Mechanistically, Gapdh enhances *il4* transcription by promoting H3K27 acetylation at its promoter, whereas pharmacological inhibition of this pathway restores basal levels. Consistently, Il4 deficiency alleviated inflammation. These findings identify a conserved, non-canonical GAPDH signaling pathway that orchestrates chronic skin inflammation and represents a potential therapeutic target.

## Introduction

Defective or disproportionate immune responses in the skin, due to a dysregulation in the immune cell function, lead to the development of inflammatory skin diseases. These diseases are generally multifactorial, and the molecular mechanisms and causes remain unclear. Conventional treatments with corticosteroids have focused on alleviating the symptoms. Nowadays, new treatments focus on the specific cytokines produced in each disease or in the enzymes involved in the signaling pathways involved in the disorders^1^. Psoriasis is a chronic skin immune-mediated disease which affects a percentage of the global population between 2–3%. It is generally characterized by a dysregulation in the keratinocyte proliferation, followed by the infiltration of immune system cells which release inflammatory cytokines. The long-term activation of the inflammatory response causes a broad damage in the skin that could affect other tissues and organs^2,3^. Atopic dermatitis is another chronic skin inflammatory disorder manifested as a recurrent, localized eczema, that results in dry skin, skin lesions and lichenification, and is usually developed in childhood. This disease is one of the most prevalent inflammatory skin disorders, with an increasing incidence between 15 and 20% in children and around 10% in adults worldwide^4,5^. While psoriasis and atopic dermatitis are distinct chronic skin inflammatory disorders characterized by TH17 and TH2/TH22 immune responses, respectively, a notable overlap has been observed in approximately 1.3% of patients diagnosed with either condition^6^.

Psoriatic patients have shown an increase in the neutrophil-to-leukocyte ratio (NLR), in the neutrophil activity, and an increase in the number of immature circulating neutrophils^7,8^. Neutrophils, together with mast cells, have been described as the main interleukin 17A (IL-17A) producers in the skin of psoriatic patients, recruiting and activating other immune cells^9,10^. Although the mechanism by which neutrophils are activated to produce IL-17A is unknown, the interaction between them and keratinocyte is a crucial process in this shift^11^. Moreover, matrix metalloproteinase 9 (MMP9) released by neutrophils in psoriasis skin increases vascular endothelial cell activation and permeability, increasing inflammation^12,13^. Similarly, recent studies are suggesting the involvement of neutrophils in the progression of atopic dermatitis. The NLR correlates significantly with the severity of the disease, being reflected in the higher area of the affected regions in the skin, and in a higher degree of inflammation in these regions^14–16^. Transcriptomic analyses in skin samples of patients with acute atopic dermatitis showed overexpression of genes related to neutrophilic inflammation, a feature of psoriasis^17^. Recent studies have shown in mice models of psoriasis and atopic dermatitis that neutrophil depletion attenuated inflammation in psoriasis^18–20^ and alleviates chronic itch in dermatitis^21^.

Glyceraldehyde-3-phosphate dehydrogenase (GAPDH) is one of the most abundant proteins in cells and is commonly regarded as a housekeeping gene. While it is well-known for its role in glycolysis, GAPDH also functions as a moonlighting protein, participating in a diverse array of non-canonical activities, some of which are linked to pathological conditions such as inflammation and cancer^22^. In oxidative stress conditions, NO synthase (NOS)-derived NO triggers the nitrosylation of GAPDH on its active site residue, the Cys^152^, generating SNO-GAPDH. Nitrosylated-GAPDH interacts with the SIAH E3 ubiquitin protein ligase 1 (SIAH1). The complex formed with SNO-GAPDH-SIAH1 translocates to the nucleus thanks to the SIAH1 nuclear localization sequence (NLS)^23–25^. Once in the nucleus, SNO-GAPDH can transfer its nitrosyl group to target proteins, functioning as a nitrosylase and altering their activities^23,25^. Additionally, it may regulate gene expression by interacting with and activating the P300/CBP complex, which facilitates the acetylation of target proteins and histones, thereby influencing gene expression^22,26–28^.

In this study, we report that the nuclear translocation of GAPDH is critical for neutrophil infiltration and activity, which in turn influences keratinocyte behavior and contributes to the pathophysiology of skin disorders. By demonstrating the intricate relationship between GAPDH and neutrophil-driven inflammation, we provide new insights that could pave the way for novel therapeutic strategies aimed at targeting these pathways in chronic skin inflammatory conditions.

## Results

### Nitrosative stress contributes to skin inflammation in the Spint1a-deficient zebrafish model

Oxidative and nitrosative stress are associated to inflammation, being peroxynitrite the most dangerous specie reported^29,30^. Taking it into consideration, we analyzed 72 hour-post-fertilization (hpf) larvae of the well-established Spint1a-deficient zebrafish model of chronic skin inflammation^31^ by immunohistochemistry using an antibody which detects nitrosylation. The results showed increased nitrosylated proteins in the skin of Spint1a-deficient larvae (Figures 1A–1B). Moreover, the higher levels of nitrosylation were predominantly observed in the keratinocyte aggregates (Figures 1C), which are associated with inflammation severity, skin hyperproliferation and oxidative stress^31^.

In order to check the impact of nitrosative stress in skin inflammation, Spint1a-deficient larvae were treated for 48 h with linsidomine chlorhydrate (SIN-1), a nitric oxide donor that generates peroxynitrite^32^. SIN-1 treatment, as expected, exacerbated inflammation, assayed as increased skin neutrophil infiltration and number of keratinocyte aggregates (Figures 1D–1F). As peroxynitrite is one of the most aggressive reactive nitrogen species (RNS), control *spint1a* +/+ larvae were also treated with SIN-1 to evaluate its toxicity. However, no significant changes were observed between the percentage of neutrophils outside the CHT of Spint1a-deficient larvae treated with DMSO or SIN-1 (Figures 1G).

We next examined the expression profile of genes involved in oxidative stress and antioxidant responses using freely available transcriptomic data from healthy subjects and psoriasis (GSE13355) and atopic dermatitis patients (GSE57225) (**Figure S1**). Among the studied genes, *NOX1–5* (NADPH oxidases), *NOS1–3*, *PTGS1–2* (prostaglan-dinendoperoxide synthases), *XDH* (xanthine dehydrogenase), *DUOX* (dual oxidase), *CAT* (catalase), *SOD1/2* (superoxide dismutases) were analyzed. *DUOX1*, *XDH*, *NOS3* and *SOD2* showed increased transcript levels in both psoriasis and atopic dermatitis lesional skin. Moreover, psoriasis patient skin showed increased mRNA levels of *NOX1* and *NOS1–2*, while atopic dermatitis patients showed higher levels of *NOX4*. In addition, antioxidant genes like *SOD1* and *CAT* were downregulated in both conditions. In addition, *SOD1*, *SOD2*, *XDH*, *CAT* and *NOS2* were the most significant differentially expressed genes in both psoriasis and atopic dermatitis among oxidative stress and antioxidant response genes investigated.

### GAPDH nuclear translocation regulates skin inflammation in zebrafish and human

The overproduction of ROS and RNS leads to an enormous cell stress that may trigger cell death by different pathways^31,33,34^. In fact, we have recently reported that, in the Spint1a-deficient larvae, there was an alteration in the NAD^+^ synthesis pathway that led to increased oxidative stress and *parthanatos* cell death^31^. As GAPDH is an NAD^+^-dependent enzyme and it has been reported that (i) it is nitrosylated and translocated to the nucleus during oxidative and nitrosative conditions, triggering a caspase-independent cell death process^26,35,36^, (ii) PARP1 can interact with GAPDH in the nucleus, leading to PARP1 activation^25,37^, and (iii) GAPDH promotes NAMPT nuclear translocation, which increases NAD^+^ levels in the nucleus^38^, we hypothesized that GAPDH is an enzyme involved in chronic skin inflammation. Accordingly, we carried out immunohistochemical staining for GAPDH in skin biopsies from healthy controls and in lesional skin samples from individuals with psoriasis. On the one hand, we observed that GAPDH was mainly found in the spinosus layer (SL) of the epidermis in biopsies from healthy subjects, with some cells presenting high levels of the enzyme in their nucleus. The expression of GAPDH in the dermis (D) was weak (**Figures S2A**). On the other hand, the psoriasis biopsies showed a robust expression of GAPDH in the epidermis along all the SL with the presence of many cells presenting the enzyme in their nucleus. Furthermore, the dermis of psoriasis patients showed GAPDH in the nucleus and in the cytosol of numerous cells (**Figures S2A)**. Notably, immune cells (CD45^+^) infiltrating the dermis of psoriatic lesions exhibited heterogeneous GAPDH expression. While the majority displayed cytoplasmic GAPDH, two distinct subpopulations were identified: one lacking detectable GAPDH expression and another characterized by predominant nuclear localization of the enzyme (**Figures S2B, S2C**). Unfortunately, it was not possible to perform a similar analysis in healthy skin due to the absence of infiltrating immune cells in these samples. Similarly, Spint1a-deficient larval skin showed increased amount of Gapdh (**Figures S3A, S3B**).

The drastic induction of GAPDH and its nuclear localization in lesional skin from psoriasis patients, together with its induction in the inflamed skin of Spint1a-deficient larvae, suggest a possible implication of GAPDH in the development and progression of this disease. Therefore, we treated Spint1a deficient larvae for 24 h with CGP3466B (Omigapil) (Figure 2A), a specific inhibitor of GAPDH nuclear translocation^39,40^ which is approved by the FDA for the treatment of congenital muscular dystrophies. CGP3466B blocks the nitrosylation of the Cys^152^ of GAPDH, avoiding its binding to SIAH1 and subsequent nuclear translocation (Figures 2B)^39,40^. Mutant larvae treated with the inhibitor showed a strong reduction of skin neutrophil infiltration and keratinocyte aggregate number (Figures 2C–2F). Notably, CGP3466B treatment did not inhibit GAPDH enzymatic activity; in fact, it increased GAPDH glycolytic activity in control larvae, while no changes were observed in Spint1a-deficient larvae (Figures 2G, 2H). In addition, wild type larvae showed slightly higher Gapdh enzymatic activity than their Spint1a-deficient siblings (Figures 2H), despite the latter having higher Gapdh protein amount (**Figures S3B**). These results were further confirmed by genetic inhibition of *siah1* which was able to ameliorate skin inflammation of Spint1a-deficient larvae, as assayed as reduced neutrophil dispersion and number of keratinocyte aggregates (Figures 2I–2K).

We next analyzed Gapdh localization by whole-mount immunohistochemistry in Spint1-deficient larvae and the results revealed a strong heterogeneity in the protein amounts of Gadph in skin cells (Figures 3A), as observed in skin biopsies from psoriasis patients. Unexpectedly, neutrophils infiltrating the inflamed skin were strongly stained with the anti-Gapdh antibody, particularly those in contact with keratinocyte aggregates (Figures 3A). Notably, treatment of Spint1a-deficient larvae with CGP3466B resulted in decreased number of dead (TUNEL^+^) (Figures 3B, 3C) and proliferating (EdU^+^) cells in the skin (Figures 3D, 3E). We next wondered whether a similar mechanism operates in human skin. To do so, human organotypic 3D skin models of psoriasis were used to determine the impact of GAPDH nuclear translocation (Figures 3F). The results showed that the inhibition of GAPDH nuclear translocation with CGP3466B was able to reduce the transcript levels of pathology-associated genes, including *S100A8*, *NAMPT* and *DEF4B* (Figures 3G–3I).

### Nuclear translocation of Gapdh, but not Gapdhs, contributes to skin inflammation in Spint1a-deficient larvae

In order to understand the role played by Gapdh nuclear translocation in skin inflammation, single cell RNA-seq (scRNA-seq) experiments were carried out in pooled tails from 3 dpf *spint1a* +/+ and *spint1a* −/− larvae. The analysis revealed a total of 34 different clusters that were grouped in 17 different mayor clusters according to the cellular type and origin (**Figure S4A**). The clusters were classified using specific cell markers as endothelial cells, neutral crest, notochord, neural precursors, mesoderm, neuromasts, proliferating cells, glial cells, neurons, fibroblasts, erythrocytes, peridermal keratinocytes, basal keratinocytes, hematopoietic stem and progenitor cells (HSPCs), macrophages, neutrophils and muscle cells (**Figure S4B**). Analysis of the 2 *gapdh* paralogs revealed that *gapdh* expression was restricted to neutrophils and muscle cells (**Figures S5A, S5B**), while *gapdhs* was rather ubiquitously expressed, with the lowest transcript levels observed in muscle cells (**Figures S5A, S5C**). Similar gene expression profiles for *gapdh* and *gapdhs* were found in Daniocell online resource^41^, also revealing robust transcript levels of *gapdh* in the liver (data not shown), which was not collected in our study. Despite this specific expression profile of each *gapdh* paralog, they showed an identity of 76.51%, similar number of amino acids (333 for Gapdh and 335 for Gapdhs) and conserved residue Cys-150 (corresponding to Cys-152 of human GAPDH) involved in nuclear translocation (**Figures S6)**.

This surprising expression pattern of *gapdh* genes in zebrafish larvae prompted us to study the contribution of each paralog to skin inflammation of Spint1a-deficient larvae. Genetic inhibition of *gapdh* by CRISPR-Cas9 technology with three different crRNAs resulted in decreased skin neutrophil infiltration and number of keratinocyte aggregates in Spint1a-deficient larvae (Figures 4A–4C). The crRNA AA targeting *gapdh* was used for all subsequent experiments, since similar results were obtained with the 3 crRNAs. In sharp contrast, genetic inhibition of *gapdhs* failed to reduce neutrophil dispersion and the number of keratinocyte aggregates. Moreover, the genetic inhibition of both *gapdh* and *gapdhs* gave similar results to the single inhibition of *gapdh* (Figures 4A–4C). Genetic edition of *gapdh* and *gapdhs* decreased their respective transcript levels, probably reflecting their degradation by nonsense-mediated mRNA decay (**Figures S7A, S7B**). In addition, genetic inhibition of *gapdh* resulted in increased transcript levels of *gapdhs* (**Figures S7B**). Western blot analysis showed a robust reduction of Gapdh(s) protein amount after the genetic inhibition of *gapdhs* alone or together with *gapdh*, while inhibition of *gapdh* alone did not significantly affect Gapdh protein amount in whole larvae (**Figures S7C**). Paradoxically, mainly *gapdh* contributed to Gapdh enzymatic activity in Spint1a-deficient larvae, since Gapdh-deficient larvae showed reduced Gapdh activity compared to their wild-type and Gapdhs-deficient siblings (**Figures S7D**).

To validate the pharmacological experiments with CGP3466B and to confirm that nuclear translocation of Gapdh exacerbates skin inflammation, we next used a *gapdh* mRNA encoding Gapdh-C150S, which was reported to impair Gapdh nitrosylation and nuclear translocation^40^. While forced expression of the mRNA encoding wild type Gapdh was able to reverse the anti-inflammatory effects of genetic inhibition of *gapdh*/*gapdhs* in Spint1a-deficient larvae, forced expression of the mRNA encoding Gapdh-C150S failed to do so (Figures, 4E), despite both mRNAs rescued Gapdh protein levels in Gapdh-deficient larvae (Figures 4F). Notably, in repeated experiments we consistently observed slightly lower Gapdh protein levels in the C150S mutant compared to the wild-type rescue, although it is unlikely that this difference prevented functional validation of the nuclear translocation defect. More importantly, when ectopically expressed in HEK293T cells, wild-type Gapdh translocated to the nucleus in response to SIN-1 treatment, whereas Gapdh-C150S failed to do so and remained cytoplasmic (Figures 4G, 4H).

### Chronic Skin Inflammation Induces Neutrophil Diversification in Spint1a-Deficient Larvae

As scRNA-seq data showed that *gapdh* was expressed in neutrophils but not in keratinocytes, and given that neutrophils contribute to chronic skin inflammation and displayed strong Gapdh expression once infiltrated into the inflamed skin of Spint1a-deficient larvae, we next focused our analysis on these cells. The neutrophil cluster of the scRNA-seq analysis from wild type and Spint1a-deficient neutrophils was selected and reclustered, obtaining a total of eight different neutrophil subclusters (**Figures S8A-S8C**). A higher number of neutrophils were obtained from the Spint1a-deficient larvae than from wild type larvae, since larvae with inflamed skin have neutrophilia^31^. In addition, wild type larvae were mainly distributed in the cluster 2, although some of them were also observed in other clusters with the exception of cluster 0 and 6, that were mainly formed by Spint1a-deficient neutrophils (**Figures S8D**).

Neutrophils subclusters were analyzed and classified according to their different gene expression profiles (**Figures S9**). Gene enrichment analysis using Gene Ontology: Biological Process showed no significant pathways associated with the cluster 1 and cluster 2. Cluster 0 showed enriched expression of genes involved in extracellular matrix and cytoskeleton organization, both associated to cell migration, such as *mmp13a* and *mmp9*. Cluster 3 included genes involved in development, such as *pmp22a* and *col5a2a*. Cluster 4 was enriched in genes associated with RNA processing, although its main markers, *myb* and *csf1rb*, are involved in hematopoiesis. Cluster 5 was enriched in genes involved in cell adhesion, such as *epcam*, while cluster 6 was integrated by proliferating neutrophils, whose main significant pathways were related to cell cycle and mitosis, such as *cdk1* and *mki67*. Finally, cluster 7 was integrated by neutrophils expressing erythroid-like genes, such as *hemgn* or *cahz*, suggesting that they were erythromyeloid progenitors.

### Neutrophils contribute to skin inflammation in Spint1a-deficient larvae

In order to evaluate the role of neutrophils in chronic skin inflammation, we firstly ablated neutrophils using the Gal4/UAS system by crossing the lines *TgBAC(mpx:GAL4-VP16)* and *Tg(UAS-E1B:NTR-mCherry)*^42,43^ so that their offspring expressed NTR-mCherry in neutrophils and *spint1a* was inhibited by microinjecting the fertilized eggs with *spint1a* crRNA/Cas9 complexes. Some of the larvae were treated from 1 to 3 dpf with metronidazole, a compound that is processed by the nitrorreductase (NTR) expressed in the neutrophils, generating a toxic compound that kills them (**Figures S10A**). Metronidazole treatment drastically ablated neutrophils (<90%) (**Figures S10B**) and this led to reduced number of keratinocyte aggregates in Spint1a-deficient larvae (**Figures S10C**). Conversely, Csf3a-induced neutrophilia led to increased number of keratinocyte aggregates in Spint1a-deficient larvae (Figures 5A–5D). Furthermore, moderate positive correlations between the number of neutrophils and skin-infiltrated neutrophils with the number of keratinocyte aggregates were observed in Spint1a-deficient larvae under basal and Csf3a-induced neutrophilia (**Figures S11A, S11B**). Moreover, skin-infiltrated neutrophils also correlated to the number of keratinocyte aggregates (**Figures S11C, S11D**). Notably, confocal microscopy revealed tight interaction of neutrophils with keratinocytes of the aggregates of the inflamed skin of Spint1a-deficient larvae (**Figures S12**).

### Nuclear translocation Gapdh in neutrophil exacerbates skin inflammation in Spint1a-deficient larvae

The relevance of neutrophils in the exacerbation of inflammation and their tight interactions with keratinocytes prompted us to study whether Gapdh nuclear translocation played a role in neutrophil pro-inflammatory activity and behavior using pharmacological and genetic inhibition approaches. Pharmacological inhibition of Gapdh nuclear translocation with CGP3466B not only resulted in reduced neutrophil dispersion and skin infiltration (Figure 2F), but also, in decreased number of neutrophils interacting with keratinocyte aggregates (Figures 5E, 5F). However, oxamate, which inhibits lactate dehydrogenase (LDH) and alleviated skin inflammation in this model (Naranjo-Sánchez, 2023), reduced the number of keratinocyte aggregates and neutrophil infiltration into the skin, but failed to reduce the number of neutrophil-keratinocyte aggregate interactions (Figures 5E, 5F). Similarly, CGP3466B was also able to reduce the number of keratinocyte aggregates in the Spint1a-deficient larvae upon Csf3a-induced neutrophilia (Figures 5G). Moreover, CGP3466B was able to alleviate neutrophilia in both cases (Figures 5H, 5I). Furthermore, genetic experiments confirm the cell-autonomous effect of Gapdh nuclear translocation in neutrophils, since overexpression of wild type Gapdh in neutrophils increased the number of keratinocyte aggregates and skin neutrophil infiltration, while the nuclear translocation mutant Gapdh-C150S failed to do so (Figures 6A–6D). Notably, the total number of neutrophils was not altered by the neutrophil-specific expression of either Gapdh (Figures 6B, 6E). Notably, immunostaining of Gapdh in Spint1a-deficient larvae revealed strong Gapdh expression in neutrophils infiltrating the skin, with nuclear Gapdh predominantly localized in euchromatin regions (Figure 6F). Furthermore, treatment with CGP3466B reduced Gapdh nuclear localization (Figures 6F–6H).

We then analyzed the behavior of skin-infiltrated neutrophil in Spint1a-deficient larvae by time-lapse confocal microscopy. It was found that neutrophils interacting with keratinocyte aggregates stayed in the same place for longer times than other neutrophils infiltrated in the inflamed skin (**Figures S13A-S13C**). Strikingly, pharmacological inhibition of Gapdh nuclear translocation with CGP3466B reduced this time of interaction, reaching the levels of neutrophils interacting with keratinocytes not involved in aggregate formation (**Figure S13B**). The speed of the neutrophils was also measured in the generated movies (**Video 1**), showing a significant reduction after the treatment with CGP3466B (**Figures S13C**). These results were further confirmed by genetic inhibition of *gapdh* that was also able to decrease the interaction time between neutrophils and keratinocyte aggregates (**Figures S13D, S13E and Video 2**).

### Nuclear translocation of Gapdh links Il4 expression in neutrophils to skin inflammation

To understand the mechanisms involved in the regulation of neutrophil by Gapdh nuclear translocation, the tail from Spint1a-deficient larvae treated with CGP3466B from 48 to 72 hpf was also analyzed by single cell RNA-seq. We found that inhibition of Gapdh nuclear translocation reduced the transcript levels of keratin 4 (*krt4*), *col1a1a*, *col1a2*, dual specificity phosphatase 5 (*dusp5*) and interleukin 4 (*il4*), while increasing those of pleckstrin homology domain containing, family F (with FYVE domain) member 1 (*plekhf1*), transcobalamin beta b (*tcnbb*), nuclear factor of kappa light polypeptide gene enhancer in B-cells inhibitor alpha a (*nfkbiaa*) and *pim2* (Figures 7A, 7B). Since the transcript levels of *nfkbiaa* increased in neutrophils of Spint1a-deficient larvae upon inhibition of Gapdh nuclear translocation, we wondered if the nuclear translocation of Gapdh was able to inhibit the activity of NF-κB, resulting in alleviation of inflammation in the *spint1a* −/− larvae. To test this, the reporter line *Tg(NF-κB:eGFP)*^44^ was used in order to measure the NF-κB-associated inflammation (Figures 7C). Significant decreased NF-κB activity was observed in the skin of Spint1a-deficient larvae after the treatment with CGP3466B (Figures 7C, 7D). In addition, a weak, but statistically significant, correlation was observed between the number of neutrophils in the skin and NF-κB activity (Figures 7E). Moreover, genetic inhibition of *gapdh* or *gapdhs* failed to reduce NF-κB activity in the skin of Spint1a-deficient larvae (Figures 7F). Furthermore, expression of a dominant negative (DN) form of Nfkbiaa^45^ in neutrophils failed to attenuate skin inflammation, assayed as the number of keratinocyte aggregates (Figures 7G, 7H), the number of neutrophils infiltrated into the skin (Figures 7G, 7I, 7J), and the total number of neutrophils (Figures 7G, 7K).

As NF-κB did not regulate neutrophil infiltration into the inflamed skin and most inflammation-related genes were similarly induced in neutrophils and keratinocytes by inhibition of Gapdh nuclear translocation, we turned our attention to *il4* because of its expression was highly enriched in neutrophil cluster 0 (Figures 8A, 8B). Interestingly, Il4 deficiency robustly alleviated the number of keratinocyte aggregates of Spint1a-deficient larvae (Figures 8C, 8D). Since cluster 0 is enriched in genes involved in actin filament reorganization and includes *mmp9* and *mmp13*, we leveraged the recently reported *mmp9* reporter line^46^. Most neutrophils infiltrating the skin and interacting with keratinocyte aggregates were *mmp9*^+^, and inhibition of Gapdh nuclear translocation with CGP3466B drastically reduced the number of *mmp9*^+^ neutrophils patrolling the skin (Figure 8E **and Video 3**).

To investigate the mechanism by which nuclear Gapdh regulates *il4* expression in activated neutrophils, and considering its potential role in gene regulation through interaction with and activation of the P300/CBP complex^22,26–28^, we performed a CUT&RUN assay to analyze histone H3 acetylation at 4 upstream regulatory regions of the *il4* gene predicted to be acetylated^47^. The results showed that both H3K27ac and Gapdh were particularly enriched in Spint1a-deficient larvae at a region near the transcription start site, and treatment with CGP3466B reduced both to basal levels (Figure 8F).

### Inhibition of Gapdh nuclear translocation reduces neutrophil recruitment during acute inflammation

We finally investigated whether Gapdh nuclear translocation may also regulate acute inflammation using a tail wounded assay^48^ (Figures 9A). The results showed that the number of neutrophils recruited to the wounded tail was considerably reduced at 5 h post-wounding (hpw) when the larvae were pre-treated for 3 h with CGP3466B (Figures 9B–9C). As CGP3466B treatment also decreased neutrophil number, it was decided to quantify the percentage of recruited neutrophils and it was confirmed that CGP3466B was able to reduce neutrophil recruitment (Figures 9D).

We next performed a dynamic study of the neutrophil migration to wound by recording the larvae for 5 h (**Video 4,** Figures 10). At 0 hpw no difference in the percentage of neutrophils recruited to the wound was observed. The difference started to be observed as soon as 1 hpw, when the DMSO-treated larvae showed a slightly increased percentage of neutrophils recruited. The differences increased over time, observing the most significant difference at 5 hpw, coinciding with the peak of neutrophil recruitment in the wound (Figures 10B, 10C). Moreover, it was also observed that while the percentage of recruited neutrophils increased from 4 to 5 hpw in DMSO-treated larvae, it no longer increased in the CGP3466B-treated group (Figures 10).

The signals that control early neutrophil recruitment are completely different to those which trigger the amplification of neutrophil recruitment, in some conditions known as “swarming”^49,50^. Therefore, we analyzed early (0–2 hpw) and late (2–5 hpw) recruitment and although it can be observed that neutrophils of CGP3466B-treated larvae showed delayed recruitment and took more time to respond to wound (**Video 4**), the difference in the displacement length of the neutrophil tracks of both groups was similar during early and late recruitment (**Figures S14**). Furthermore, neither the speed nor the displacement length of random migration of neutrophils in the head of the larvae were altered after the treatment with CGP3466B, indicating that Gapdh nuclear translocation does not regulate neutrophil random migration (**Figures S15, Video 5**).

## Discussion

The relevance of nuclear GAPDH in inflamed skin was demonstrated for the first time using well established zebrafish models of chronic and acute inflammation (**Figure S16**). Pharmacological and genetic approaches showed that Gapdh nuclear translocation not only reduced the number and percentage of neutrophils infiltrated in the skin but also the keratinocyte aggregate number, hyperproliferation and cell death. Gapdh did not alter neutrophil random migration, suggesting that Gapdh nuclear translocation specifically regulates neutrophil migration in the context of inflammation. Importantly, pharmacological inhibition of Gapdh nuclear translocation with CGP3466B did not affect Gapdh enzymatic activity, demonstrating that Gapdh nuclear translocation, rather than its enzymatic activity, is promoting skin inflammation. Furthermore, Gapdh activity was found to be lower in Spint1a-deficient larvae than in their wild type siblings, probably reflecting the inhibition of Gapdh enzymatic activity by oxidative and nitrosative stress and its subsequent nuclear translocation. These results were further confirmed by the genetic inhibition of *siah1* in the zebrafish model and by pharmacological inhibition of GAPDH nuclear translocation with CGP3466B in human organotypic 3D psoriasis models^31,51–53^. We also observed that inhibition of Gapdh nuclear translocation with CGP3466B led to decreased Gapdh protein levels in Spint1a-deficient larvae. Further experiments are required to understand whether inhibition of Gapdh nuclear translocation promoted its degradation, the mechanism involved and its relevance on its canonical function. Whatever the outcome, our results pointed to GAPDH nuclear translocation as an important driver of chronic skin inflammation and as a promising target for therapeutical intervention in chronic skin inflammatory disorders.

The role played by GAPDH in the nucleus may have clinical relevance since immunohistochemical analysis of skin biopsies from psoriasis patients revealed the overproduction of GAPDH in the inflamed skin. Strikingly, although control subjects showed some keratinocytes in the epidermis with high levels of GAPDH in their nucleus, which may suggest a physiological role of GAPDH in keratinocytes, GAPDH was hardly detected in healthy dermis. In sharp contrast, GAPDH was robustly accumulated in both the nucleus and cytosol of epidermal keratinocytes of psoriasis lesional skin and in the nucleus of several cells of the dermis. It is tempting to speculate that these cells are infiltrating immune cells that translocated GAPDH upon hyperactivation. Although further experiments are required to confirm this motivating and ambitious hypothesis, our results suggest that the translocation of GAPDH to the nucleus may be used as a diagnostic biomarker in psoriasis. Given that CGP3466B (Omigapil) is an FDA-approved drug and has received orphan designation by the EMA for the treatment of congenital muscular dystrophies, it holds significant promise as a repurposed therapeutic candidate for chronic inflammatory diseases.

Our scRNA-seq study is that *gapdh*, which was supposed to be the main one, was only expressed in neutrophils and muscle cells of 3 dpf larval tail, while *gapdhs* was rather ubiquitously expressed, except for muscle cells, where their transcript levels were much lower than in the rest of the tissues. Similar gene expression profiles of *gapdh* and *gapdhs* were found in Daniocell online resource^41^, also revealing robust transcript levels of *gapdh* in the liver and pancreas, which were not collected in our study. The identity of both proteins is 76,51% and they showed key residues involved in non-canonical functions, including the C152 (C150 in zebrafish), involved in nuclear translocation; G10, involved in nuclear GAPDH-PARP1 interaction; and Y41, involved in GAPDH phosphorylation, among others. In human, GAPDH has a molecular mass of 36 kDa and contains 335 amino acids, while GAPDHS has a molecular mass of 44,5 kDa and contains 408 amino acids, with a specific 72-amino acid length N-terminus^54,55^. Loss- and gain-of-function experiments using CRISPR-Cas9 (wild type and nuclear translocation-deficient C150S Gapdh, respectively, confirmed that Gapdh, but not Gapdhs, nuclear translocation, rather than its canonical glycolytic activity, promotes chronic skin inflammation in the Spint1a-deficient model.

One important observation from our study is that the number of neutrophils infiltrated in the skin of Spint1a-deficient larvae positively correlated with skin inflammation and, more importantly, manipulation of neutrophil numbers using two different experimental approaches affected skin inflammation. This result contrasts the inability of neutrophil ablation using a morpholino against the gene encoding the master myeloid transcription factor Spi1 (also known as Pu.1) to reduce keratinocyte aggregates in this model^56^. The discrepancy could be explained by the different experimental approaches used, since genetic inhibition of *spi1* would also result in macrophage ablation and the role of this cell type in the skin inflammation of this model is unknown. However, both studies demonstrate that neutrophils are not causative of epidermal defects but rather exacerbated it. Unexpectedly, we observed that neutrophils can divide into the inflamed skin. This process has been observed to take place near the keratinocyte aggregates, where neutrophils stop, acquire a spherical shape, then divide producing two different new neutrophils that continue migrating through the skin. Conversely, our results also suggest that neutrophils may release factors that exacerbate keratinocyte proliferation and skin inflammation.

It is complicated to envisage the molecular mechanism orchestrating neutrophil behavior and pro-inflammatory activity by nuclear Gapdh because the available information on the non-canonical functions of Gapdh in neutrophils is rather limited. Our scRNA-seq study revealed that Gapdh nuclear translocation regulates *il4* gene expression in neutrophils of Spint1a-deficient larvae which, in turn, aggravated skin inflammation. IL4 is an anti-inflammatory cytokine mainly produced by basophils that has been shown to regulate B cells, ILCs, macrophage polarization to M2 and T cell polarization to T_H_2^57^. We found that in larvae with inflamed skin, *il4* transcript levels were dramatically induced in neutrophils, and pharmacological inhibition of Gapdh nuclear translocation robustly attenuated them. The expression of *IL4* in mammalian neutrophils was also reported in a few studies^58,59^. Importantly, Il4 deficiency alleviated skin inflammation of Spint1a-deficient larvae, assayed as the number of keratinocyte aggregates. This is not surprising, since the IL-4Rα has been broadly studied in atopic dermatitis disease, where T cells and keratinocytes were both found to express it and to respond to IL-4^60–62^. In addition, IL-4 induces the expression of IL-4Rα in keratinocyte, resulting in a positive feedback loop that amplifies inflammation^61^. In agreement with these results, epidermal keratinocytes expressed *il4r.1* and *il4r.2*, according to the Daniocell web resource^41^, suggesting that the Il4 produced by neutrophils might directly regulate keratinocyte hyperproliferation and skin inflammation. Our findings strongly support this notion, as we observed that a subset of *mmp9*^+^ activated neutrophils express *il4* and that these *mmp9*^+^ neutrophils predominantly interact with keratinocyte aggregates in inflamed skin.

Pharmacological inhibition of Gapdh nuclear translocation regulated the expression of several inflammation-related genes in neutrophils, such as *nfkbiaa*, which encodes an inhibitor of NF-κB^63^. Our functional study showed that pharmacological inhibition of Gapdh nuclear translocation in Spint1a-deficient larvae resulted in reduced NF-κB activity in the skin. This is not unexpected, since GAPDH was reported to act as an enhancer of NF-κB activity in non-Hodgkin’s B lymphoma and in human angioimmunoblastic T cell lymphoma^64,65^. Furthermore, inhibition of GAPDH/SIAH1 nuclear translocation with sivelestat sodium inhibits progression of spinal cord injury by decreasing the activation of NF-κB^66^. Similarly, we found that inhibition of Gapdh nuclear translocation with CGP3466B increased the levels of *nfkbiaa*, leading to decreased NF-κB activity, indicating that Gapdh nuclear translocation triggered NF-κB activity. Nevertheless, genetic inhibition of NF-κB in neutrophils failed to alleviate skin inflammation of the Spint1a-deficient larvae. These results suggest, therefore, that although Gapdh nuclear translocation activates NF-κB in neutrophils, this did not contribute to the progression of chronic skin inflammation. However, as inhibition of Gapdh nuclear translocation showed reduced skin NF-κB activity, pharmacological modulation of Gapdh nuclear translocation could be a potential treatment in inflammatory diseases where NF-κB has been found to be directly involved on their progression, such as atherosclerosis, inflammatory bowel disease, rheumatoid arthritis, psoriasis and atopic dermatitis, among others^67,68^.

Another notable finding of our study is the molecular mechanism by which Gapdh regulates *il4* and other inflammation-related genes. While we cannot rule out the possibility that GAPDH modulates these genes by binding to the 3’UTR of their mRNAs^69,70^, especially since inhibiting Gapdh nuclear translocation leads to its cytosolic accumulation, our results indicate that Gapdh localized in euchromatin region of neutrophil nucleus and may also directly regulate *il4* expression by enhancing H3K27 acetylation at its promoter. This effect is likely mediated through its interaction with and activation of the P300/CBP complex, as previously demonstrated in mammals^22,26–28,39,40^.

In summary, our study identifies GAPDH nuclear translocation as a critical driver of chronic skin inflammation, demonstrated using zebrafish models and validated in human psoriasis models. We show that GAPDH translocation, rather than its enzymatic activity, promotes inflammation by inducing *il4* gene expression in neutrophils via acetylation of H3K27 at its promoter, which exacerbates keratinocyte hyperproliferation and skin inflammation. Immunohistochemical analysis of psoriasis patient biopsies revealed increased GAPDH nuclear localization in lesional skin, supporting its relevance as a biomarker and therapeutic target. Although we attempted to reproduce GAPDH nuclear translocation in purified human neutrophils stimulated *in vitro*, we did not observe consistent results, likely reflecting the requirement of a complex *in vivo* inflammatory context. As CGP3466B (Omigapil) is an FDA- and EMA-approved compound, it represents a highly promising therapeutic candidate for repurposing to effectively treat chronic inflammatory diseases.

## Materials and Methods

### Ethics statement

Experiments and procedures were performed as approved by the Bioethical Committees of the University of Murcia (approval numbers #669/2020) and Ethical Clinical Research Committee of The University Hospital Virgen de la Arrixaca (approval number #8/13). The experiments performed with animals comply with the Guidelines of the European Union Council (Directive 2010/63/EU) and the Spanish RD 53/2013. The studies with human samples were carried out following the principles expressed in the Declaration of Helsinki, as well as in all the other applicable international, national, and/or institutional guidelines for the use of samples and data. All human samples were obtained from the Biobank of IMIB-Pascual Parrilla with registration number #B.0000859.

### Animals

Wild-type zebrafish (*Danio rerio*) were obtained from the Zebrafish International Resource Center (ZIRC). The handling was carried out according to the zebrafish handbook rules^71^. The different zebrafish transgenic lines used in this project were *Tg(lyz:dsRED)*^nz50 72^, *Tg(NFkB-RE:eGFP)*^*sh235Tg* 44^, *Tg(mpeg1:eGFP)*^gl22 73^, *Tg(mpx:Gal4.VP16)*^*i222* 43^, *Tg(UAS:nfsB-mCherry)*^*c264* 43^, *Tg(lyz:BFP)*^74^, *Tg(lyz:MITO-Dendra2)*^75^, *Tg(−6.0itga2b:eGFP)*^*la2*^ *[Tg(cd41:GFP) for clarity]*^76^*, Tg(gata1a:dsRED)*^*sd2* 77^ and *spint1a*^*hi2217Tg/hi2217Tg*^ (*spint1a* mutant for simplicity)^56^.

### Chemical treatments in zebrafish

Zebrafish embryos were subjected to manual dechorionation at 24 hpf. Larvae were treated from 24 hpf to 48 hpf or 72 hpf, or from 48 hpf to 72 hpf by chemical bath immersion at 28°C. The incubation was carried out in 6-well plates containing 20 larvae per well in egg water (60 μg/mL of sea salts in distilled water) with methylene blue. As all the compounds were diluted in dimethyl sulfoxide (DMSO), the control larvae wells were supplemented with 1% DMSO. The different compound used and their characteristics are shown in the **Table S1**.

For neutrophil ablation, *Tg(mpx:Gal4.VP16; UAS:NTR-mCherry)* larvae were treated at 1 dpf with 5 mM Mtz and kept in dark. At 48 hpf the drug was renewed, and larvae were treated up to 3 dpf. The larvae were imaged once a day up to 7 dpf and the number of neutrophils determined^43,78^.

### Gain- and loss-of-function experiments in zebrafish

The CRISPR RNA (crRNA) obtained from Integrated DNA Technologies (IDT) with the following target sequence were used: *gapdh* AA crRNA: 5’- AAGACCGTTGATGGGCCCTC-3’; *gapdh* AB crRNA: 5’- TTCCATGGGTGGAGTCGTAC-3’; *gapdh* AD crRNA: 5’- GGACCCAGCCAATATTAAGT-3’; *gapdhs* crRNA: 5’- TCCCTTGTAACGGCCATGAG-3’; *siah1* crRNA: 5’- AATTACGGATCGACCCAAGC-3’ and *spint1a* crRNA: 5’- TACGAATGGGAGCAAGTGGTCGG-3’. They were resuspended in duplex buffer at 100 μM, and 1 μL was incubated with 1 μL of 100 μM trans-activating CRISPR RNA (tracrRNA) at 95°C for 5 minutes and then 5 minutes at room temperature (RT) to form the complex. Then, the mix was diluted with 1.45 μL of duplex buffer. One μL of this complex was mixed with 0.30 μL of recombinant Cas9 (10 mg/mL, IDT), 0.25 μL of phenol red, and 2.55 μL of duplex buffer. When two crRNAs were microinjected at the same time, they were incubated with the tracrRNA independently, and 1 μL of each complex was mixed with 0.30 μL of recombinant Cas9 (10 mg/mL), 0.25 μL of phenol red, and 1.55 μL of duplex buffer. The efficiency of the *siah1* crRNA was determined by analyzing its ability to reverse skin inflammation of Spint1a-deficient larvae, since we were unable to amplify the target region and no cross-reactive antibody was available for zebrafish. In the case of the *gapdh* and *gapdhs* crRNAs, due to issues in the sequencing of the target sequence, the efficiency was determined by western blot, RT-qPCR and Gapdh activity assays. Spint1a crispants were easily identifiable based on their well-characterized phenotype, which closely resembles that of Spint1a-deficient mutants. Specifically, these larvae exhibited keratinocyte aggregates and neutrophil infiltration in the skin, both of which are hallmark features of the inflammatory skin phenotype associated with spint1a loss of function. Crispant larvae of 3 dpf were used in all studies.

In vitro-transcribed RNA was obtained following the manufacturer’s instructions (mMESSAGE mMACHINE kit, Ambion). RNAs were mixed in microinjection buffer and microinjected into the yolk sac of one-cell-stage embryos using a microinjector (Narishige) (0.5–1 nL per embryo). The same amount of RNA was used in all experimental groups.

The *lyz:gapdh* and *lyz:gapdh*^*C150S*^ constructs were generated by MultiSite Gateway assemblies using LR Clonase II Plus (Life Technologies) according to standard protocols and using Tol2kit vectors described previously^79^. The corresponding transgenic lines were generated by microinjecting 1 nl of a solution containing 38.5 ng/μl of the respective plasmids, 50 ng/μl of Tol2 transposase RNA, and 0.05% phenol red into the yolk sac of one-cell-stage embryos using a microinjector (Narishige).

### Zebrafish larvae imaging with stereomicroscope

Forty-eight and 72 hpf larvae were anesthetized using buffered tricaine at 200 μg/mL dissolved in egg water supplemented with methylene blue. Images were captured with an epifluorescence LEICA MZ16FA stereomicroscope set up with green and red fluorescent filters. All images were acquired with the integrated camera on the stereomicroscope and were analyzed to determine the number of neutrophils and their distribution in the tail with the zebrafish line *Tg(lyz:dsRED2)* and the number of keratinocyte aggregates, which are readily visible under bright-field microscopy as dense clusters of keratinocytes in the outer layers of the skin, using a ROI from the anal opening to the tip of the tail^31^. To estimate neutrophil infiltration into the skin, we primarily quantified the percentage of neutrophils located outside the caudal hematopoietic tissue (CHT). While in wild-type larvae the vast majority of neutrophils (~90%) remained within the CHT at 2–3 dpf, in Spint1a-deficient larvae approximately 40% were found outside this region, indicating active skin infiltration^31^. The transcriptional activity of NF-κB was visualized and measured with the zebrafish line *Tg(NFκB-RE:eGFP)* using a ROI located in skin^31^.

### WIHC in zebrafish

Embryos/larvae were directly fixed in 4% paraformaldehyde (PFA). Embryos/larvae were then washed with phosphate buffer saline (PBS) with 0.1% tween-20 (PBST) 3 times for 5 minutes. In order to dehydrate the sample progressively, 25%/75% methanol (MeOH)/PBST, 50%/50% MeOH/PBST and 75%/25%MeOH/PBST and 100% MeOH were employed each for 5 minutes. At this point, embryos were stored at −20°C. To proceed with the immunofluorescence, samples were hydrated in decreasing solutions of MeOH/PBST, as previously described, and then washed 3 times for 5 minutes with PBST. The following primary antibodies were used: rabbit anti-GAPDH (Abcam, #ab181602, 1:500), rabbit anti-Nitrotyrosine (Thermofisher, #A-21285, USA, 1:200). Secondary antibodies were goat anti-rabbit Alexa Fluor 488 (#A-11008, 1:1,000) and Alexa Fluor 594 (#A-11012, 1:400). Samples were then stained with a DAPI solution (1:1,000) in PBST for 20 minutes followed by a wash out step of 3 times for 10 minutes with PBST. Finally, embryos were transferred to antifade mounting medium Vectashield (VECTOR, #H-1000) and stored in the dark at 4°C until imaging. To determine the nuclear localization of Gapdh, images were taken using a STELLARIS 8 FALCON-STED (LEICA) confocal microscope in a 2,048 × 2,048-pixel format with Zoom 6.37 and an objective HC PL APOCS2 63x/1.30 GLYC. The pinhole value was 1, corresponding to 102.87 μm. The mean fluorescence intensity of the nuclear and cytosolic compartments was measured using ImageJ software. The red (neutrophil) and blue (DAPI) channels were used to create ROIs for the cytoplasmic and nuclear compartments, respectively. No colocalization algorithm was used; instead, nuclear and cytosolic compartments were independently segmented and quantified. Additionally, representative images of Gapdh immunolocalization in the tails of spint1a mutant larvae were obtained using a Leica Thunder-TIRF imager Widefield Microscope in 1024 × 1024-pixel format and a HC PL FLUOTAR 10x/0.32 DRY objective. The total Z range was 64 μm with a step size of 4 μm. All images were subjected to Lightning Adaptive Deconvolution with the default settings of the Leica software. Proliferation in whole larvae was measured using 5-ethynyl-2’-deoxyuridine (EdU) staining. Larvae were incubated in 10 μM EdU dissolved in embryo medium for 6 hours, euthanized and fixed in 4% PFA overnight at 4°C, and stored in methanol at −20 °C until staining. Click-iT EdU Imaging Kit (Life Technologies) were used for staining following manufacturer’s instructions.

### Confocal microscopy imaging of live zebrafish larvae

All imaging was performed using a zWEDGI device^80^. Briefly, an anesthetized larva was loaded into a zWEDGI chamber for time-lapse imaging. The loading chamber was filled with 1% low melting point agarose (Sigma-Aldrich) in E3 to retain the larvae in the proper position. Additional E3 supplemented with 0.16 mg/ml tricaine was added as needed to avoid dryness and provide required moisture to zebrafish larvae during imaging acquisition. All images were acquired on a spinning disk confocal microscope (CSU-X; Yokogawa) with a confocal scanhead on a Zeiss Observer Z.1 inverted microscope equipped with a Photometrics Evolve EMCCD camera, and an EC Plan Neofluar NA 0.3/10 x air objective, z-stacks, 5 μm optical sections and 512 × 512 resolution. For whole-larvae imaging, 7 × 1 tile images were taken and automatically stitched. For time-lapse movies of tailfin injury and neutrophil and chemotaxis, images were taken every 2 minutes up to 5 hours post-wounding. For tail wound experiments, the 10X objective was used. For neutrophil behavior in *spint1a* −/− larvae, the 40X objective was used. Moreover, transgenic zebrafish larvae were also examined under a Stellaris confocal microscope with a 63× objective and further processed with Leica software to evaluate the interaction between neutrophils and keratinocytes.

To quantify the number of neutrophils, 3 dpf larvae were fixed and tailfin images were acquired as described previously. For quantification of the number of neutrophils in the wounded regions, acquired zebrafish tail images were reconstructed on IMARIS Bitplane software (Version 9.5/9.6) rendering mode and total number of neutrophils were automatically counted in the wound at various time points (0, 1-, 2-, 3-, 4- and 5 hours post wound) using IMARIS spots function. Spots were defined as particles with 5 μm and 10 μm of X/Y and Z diameter, respectively. Cells were automatically counted using the IMARIS spots function and defined as particles with 5 μm and 10 μm of X/Y and Z diameter, respectively. Cell tracking was performed in the field of view (FOV) and in the wounded area. To analyze the neutrophils interacting with the keratinocyte aggregates in the skin, a surface for each region/area representing each aggregate was created, then a mask for the neutrophil signals were generated setting to “zero” signal outside the surface. Mean neutrophil speed and displacement length were obtained by IMARIS cell tracking analysis. IMARIS volume rendering mode was used to obtain representative 3D reconstructions that were used for **Figures** and supplemental movies.

To quantify nitrosylation, the 488 nm channel images were analyzed in ImageJ. A broad ROI outlining the skin was drawn to capture overall background nitrosylation, and individual ROIs tightly surrounding keratinocyte aggregates were created to test whether those aggregates exhibited enhanced signal. Using the ROI Manager, “Set Measurements” was conFigured to record mean gray value, and the “Multi Measure” function was applied to extract average fluorescence intensity for each ROI. Intensity profiles along each aggregate ROI were exported and plotted in R with ggplot2, with the x-axis corresponding to position within the aggregate and the y-axis to nitrosylation intensity, thus enabling visualization and comparison of spatial enrichment of nitrosylation in keratinocyte aggregates versus adjacent non-aggregate regions.

### Western blot

Zebrafish embryos at 72 hpf were anesthetized in buffered tricaine (200 μg/mL) dissolved in egg water, and the end of the fin fold was amputated with a scalpel. Tissues collected from around 40 embryos were pooled and then spun and resuspended in 200 μL of 10 mM Tris pH 7.4 + 1% SDS. Samples were then incubated at 95°C for 5 minutes with 1,400 rpm agitation, followed by maximum speed centrifugation for 5 minutes. Supernatants were frozen at −20°C until proceeding. BCA kit was employed to quantify protein using BSA as a standard. Fish lysates (20 μg) in SDS sample buffer were subjected to electrophoresis on a polyacrylamide gel and transferred to PVDF membranes. The membranes were incubated for 1 hour 30 minutes with Tris Buffered Saline, with Tween 20, pH 8.0 (TTBS) containing 5% (w/v) skimmed dry milk powder and immunoblotted in the same buffer 16 hours at 4°C with the rabbit monoclonal antibody to human GAPDH (1/2500, Abcam, #ab181602), and the rabbit polyclonal anti-beta actin (1:2500, Santa Cruz Biotechnology, #sc-47778). The blot was then washed with TTBS and incubated for 1 hour at RT with secondary HRP-conjugated antibody diluted 2,500-fold in 5% (w/v) skimmed milk in TTBS. After repeated washes, the signal was detected with the enhanced chemiluminescence reagent and ChemiDoc XRS Bio-Rad.

### Analysis of gene expression

Total RNA was extracted from whole larvae or human cell pellets with TRIzol reagent (Invitrogen) following the manufacturer’s instructions and treated with deoxyribonuclease I, amplification grade (RNA, 1 U/mg; Invitrogen). SuperScript IV RNase H Reverse Transcriptase (Invitrogen) was used to synthesize first-strand cDNA with random primer from 1 μg of total RNA at 50°C for 50 min. Real-time PCR was performed with an ABIPRISM 7500 instrument (Applied Biosystems) using SYBR Green PCR Core Reagents (Applied Biosystems). Reaction mixtures were incubated for 10 min at 95°C, followed by 40 cycles of 15 s at 95°C, 1 min at 60°C, and, last, 15 s at 95°C, 1 min 60°C, and 15 s at 95°C. For each mRNA, gene expression was normalized to the *rps11* (zebrafish) or *ACTB* (human) content in each sample, using the Pfaffl method^81^. The primers used are shown in **Table S2.** In all cases, each PCR was performed with triplicate samples and repeated at least with two independent samples.

### GAPDH activity assay

Zebrafish embryos at 3 dpf were anesthetized in buffered tricaine (200 μg/mL) dissolved in egg water, and the end of the fin fold was amputated with a scalpel. Tissues collected from around 40 embryos were pooled and then spun and processed following the manufacturer’s instructions (Abcam, Human GAPDH Activity Assay Kit II, #ab284542). Gapdh activity was revealed by measuring absorbance at 450 nm using a plate reader POLARstar *Galaxy*. The absorbance at 450 nm correlates with the formation of an intermediate product between Glyceraldehyde 3-Phosphate (GAP) and biphosphoglycerate (BPG).

### Tissue dissection and single cell RNA-seq analysis in zebrafish

Around 100 zebrafish embryos at 3dpf of each group were anesthetized in buffered tricaine (200 μg/mL) dissolved in egg water, and the fin fold was amputated at the end of the yolk sac extension using a scalpel. Tissues were pooled and resuspended in PBS with liberase 1:65/volume of PBS (Roche, #05401119001), and incubated at 28°C for cell disaggregation. After processing, cell concentration was adjusted to 1000 cells/μL. The scRNA-seq libraries using the 10x Genomics’ Chromium Single Cell 3′ V2 chemistry and processed using the 10x Cell Ranger pipeline. Libraries were sequenced by Novogene.

The files generated with Cell Ranger were processed in R using Seurat package (version 4.3.0.1)^82^. Gene counts from Cell Ranger were imported using the function CreateSeuratObject, with a min.cells = 25 and min.genes =250. Low-quality cells were removed using the following thresholds: a minimum of 250 and maximum of 2500 genes, a maximum of 10% of mitochondrial gene UMIs (13 mitochondrial genes considered in total) and a maximum of 15000 UMIs in total. JackStraw function^83^ was used to predict and remove the cell doublets present in the single cell RNA-Seq. SCT transform normalization, which applies a regularized negative binomial regression and variance stabilization, was applied to the filtered dataset using the SCTransform function in Seurat^84,85^. Data were normalized and scaled. Next, principal component analysis was carried out using the RunPCA function in Seurat, followed by a cell-clustering analysis with the functions FindNeighbours and FindClusters, both with a resolution = 0.5. UMAP and TSNE dimensional reduction was performed using the RunUMAP and RunTSNE function in Seurat (reduction.use = “pca”, dims = 1:20). Cell clusters were identified and classified using the biomarkers obtained after applying the FindAllMarkers function, with a min.pct of 0.2 and using Wilcox statistic test. Despite the SCT transform, the assay data used was always the “RNA” assay. UMAP, feature plots and heatmaps were created using the DimPlot, FeaturePlot and DoHeatmap funcitions in Seurat. For the DotPlots and BarPlots generated, the package ggplot2 was used^86^. To work individually with the different populations of neutrophils, the different clusters were isolated using the subset function, and were reclustering following the previous steps carried out with the global population.

To assess the effect of the GAPDH inhibitor in the Spint1a-deficient model, we incorporated a new sample treated with the inhibitor (Spint1a-deficient CGP) into the previously established single-cell atlas. Rather than reclustering everything de novo, we used Seurat’s anchor-based label transfer framework to map the new cells onto the existing reference. Specifically, we treated the original spint1a_annotated object as the reference, computed transfer anchors between it and the inhibitor-treated query subset, and applied TransferData to infer the most likely cluster identity for each query cell along with a confidence score. We then projected the query cells into the reference UMAP space via MapQuery, allowing direct visualization of how the treated cells align with or deviate from preexisting clusters. Reference and mapped query cells were merged into a single Seurat object with a unified identity (using the transferred labels for the query), enabling side-by-side comparison of distributions.

To determine whether the inhibitor induced novel transcriptional states rather than simply fitting into the existing clusters, we also performed a joint integration of neutrophils from the reference and the treated sample. This was done by identifying integration anchors with FindIntegrationAnchors, integrating the data with IntegrateData, and then applying the same downstream dimensionality reduction (PCA, UMAP) and clustering parameters as in the original analysis. Emergence of clusters enriched for inhibitor-treated cells or cells with low label-transfer confidence flagged potential treatment-specific states for further investigation.

### Cleavage Under Target and Release Using Nuclease (CUT&RUN) in zebrafish

Zebrafish embryos at 72 hpf were anesthetized in tricaine (200 μg/mL) dissolved in egg water, and the tails were amputated with a scalpel, starting at the cloaca. Tails collected from around 60 embryos were pooled and then spun and resuspended in 1 mL PBS. Liberase at 1:65/volume of PBS (Roche, cat # 05401119001) was added, and tissues were incubated at 28°C for 35 minutes, pipetting up and down every 5 minutes. To stop the reaction, FBS was added to a final concentration of 5% in PBS. Disaggregated fin folds were filtered through a 40 μM filter and washed using PBS + 5% FBS. Cell suspension was centrifuged at 650xg for 5 minutes and resuspended in 50 μL of PBS + 5% FBS. Trypan Blue was applied to calculate cell density. After tissue dissociation, CUT&RUN was performed using CUT&RUN Assay Kit (Cell Signaling #86652)^47^. Briefly, each CUT&RUN reaction contained 100,000 cells, which were permeabilized using 2,5% digitonin. The specific antibodies Rabbit Polyclonal IgG GAPDH (Abcam, ab181602) and anti-Histone H3 antibody (Acetyl-K27) (Abcam, ab4729) were used (6 μl/each reaction). Reactions for the positive control Tri-Methyl-Histone H3 (Lys4) (C42D8) rabbit mAb (2 μl/ reaction; 1:50; Cell Signaling, #9751) and the negative control rabbit (DA1E) mAb IgG XP^®^ Isotype Control (1:20; 5 μl/reaction; Cell Signaling #66362) were included. Antibodies were incubated over night at 4°C. We followed manufactureŕs protocol “Digestion of CUT&RUN Input DNA by Mnase” to process input DNA samples. DNA from input and enriched chromatin samples were purified by DNA Purification Buffers and Spin Columns (ChIP, CUT&RUN, CUT&Tag) (Cell Signaling #14209). For quantification of DNA by qPCR, 2.5 μl of the appropriate DNA sample (diluted 1:2) to each well (10 μl final volumen) of the PCR plate was added and triplicates for each PCR reaction was performed. Real-time PCR was performed with a QuantStudio^™^ 5 Flex Real Time PCR System (Applied Biosystems) using power SYBR Green PCR Master Mix (Applied Biosystems). The reaction mixtures were incubated for 10 min at 95°C, followed by 40 cycles of 15 s at 95°C, 1 min at 60°C, and finally 15 s at 95°C, 1 min 60°C and 15 s at 95°C. Among the four promoter regions of the il4 gene predicted in silico as binding sites for the P300/CBP complex using a previously reported workflow^47^, one was experimentally confirmed using the primer pair 5’-CACGTTTGCTGTTCTCTGAAAC-3’ and 5’-AAAATGTCATGCACGCCCTC-3’. Quantitative PCR results were analyzed using a template and % input and Assay Site IP Fold Enrichment were calculated (referred in Figure as ‘Normalize to IgG’).

### Gene Expression Omnibus (GEO) database analysis

Human psoriasis (accession number: GSE13355) and atopic dermatitis (GSE57225) transcriptomic data were collected in the GEO database (https://www.ncbi.nlm.nih.gov/geo/) and analyzed in R programming language (http://www.r-project.org) using the R environment Rstudio (http://www.rstudio.com) and the Biobase, GEOquery and limma libraries. Gene expression plots were obtained using GraphPad Prism Software.

### Immunohistochemistry in human skin samples

Skin biopsies from healthy donors (n = 4) and psoriasis patients (n = 4) were used. Clinical inclusion criteria were a psoriasis diagnosis evaluated by 2 dermatologists independently and the histology of skin lesions compatible with psoriasis/psoriasiform dermatitis evaluated by a pathologist. Clinical exclusion criteria were a diagnosis compatible with other dermatoses (atopic dermatitis, lichen planus, phytodermatosis and allergic contact eczema) and histology compatible with lichenoid dermatitis/lichen planus or spongiotic dermatitis. Samples were fixed in 4% PFA, embedded in Paraplast Plus, and sectioned at a thickness of 5 μm. After being dewaxed and rehydrated, the sections were incubated in 10 mM citrate buffer (pH 6) at 95°C for 30 minutes and then at RT for 20 minutes to retrieve the antigen. Afterward, steps to block endogenous peroxidase activity and nonspecific binding were performed. Then, sections were immunostained with rabbit monoclonal antibody to GAPDH (Abcam, ab181602, 1:500) followed by 1/100 dilution of biotinylated secondary antibody followed by ImmunoCruz goat anti-rabbit Alexa Fluor 594 (Thermo Fisher Scientific, #A-11012, 1:400). Finally, after DAB staining solution was added, sections were dehydrated, cleared, and mounted in Neo-Mount or directly stained with DAPI solution as indicated above. No staining was observed when primary antibody was omitted. Sections stained with DAB were finally examined under a Leica microscope equipped with a digital camera Leica DFC 280, and the photographs were processed with Leica QWin Pro software. Sections stained with fluorescence were examined under a Stellaris confocal microscope with a 63× objective and further processed with Leica software.

For double immunofluorescence staining, sections were deparaffinize and rehydrate before performing antigen retrieval with Tris/EDTA buffer (10 mM Tris base, 1 mM EDTA solution, 0.05% Tween 20, pH 9.0). Then, they were blocked with 5% BSA in PBS for 30 mins at RT and washed with PBS, incubated with a rabbit anti-GAPDH antibody (1:1000, Abcam, ab181602) and a monoclonal mouse anti-human CD45 antibody (1:1000, Dako, M0701), followed by donkey anti-rabbit IgG H&L Alexa Fluor^®^ 488 (Thermo Fisher, # A21206) and goat anti-mouse IgG H&L Alexa Fluor^®^ 594 (Thermo Fisher, # A11032), both at 1:400, in incubation buffer (PBS + 1% BSA + 0,3% Triton X-100). Sections were then washed with PBS and incubated with DAPI (1:5000; Sigma-Aldrich, 32670), 5–7 min at darkness. Finally, they were mounted using VECTASHIELD^®^ Antifade Mounting Medium with DAPI (Vector Laboratories, H-1200). Sections were examined under a confocal microscope (Leica STELLARIS 8) and further processed with Leica software and ImageJ. The objective used was x63. The percentage of CD45+ cells negative for GAPDH, with cytoplasmic GAPDH (c-GAPDH) and with nuclear GAPDH (n-GAPDH) were manually counted in three random regions of the epidermis from each of the three biopsies analyzed. No staining was observed when primaries antibodies were omitted.

### Human organotypic 3D models of psoriasis

Insert transwells (Merck, MCHT12H48) were seeded with 10^5^ human foreskin keratinocytes (Ker-CT, ATCC CRL-4048) on the transwells in 300 μL CnT-PR medium (CellnTec) in a 12 well format. After 48 hours, cultures were switched to CnT-PR-3D medium (CELLnTEC, Bern 3014, Switzerland) for 24 hours and then cultured at the air– liquid interface for 17 days. From day 12 to 17 of the air–liquid interphase culture, the Th17 cytokines IL17A (30 ng/mL) and IL22 (30 ng/mL) were added^51^. CGP3466B (10 μM) was applied from day 14 to 17 d and the tissues were harvested for gene expression analysis at 17 d.

### Cellular localization of zebrafish Gapdh

HEK293T cells (CRL-11268; American Type Culture Collection) were maintained in DMEM:F12 (1:1) supplemented with 10% FCS, 2 mM Glutamax, and 1% penicillin–streptomycin (Thermo Fisher Scientific). They were tested for mycoplasma contamination and authenticated by STR profiling. Plasmid DNA was prepared using the Mini-Prep procedure (Qiagen). The plasmids encoding zebrafish Gapdh (wild type and C150S mutant) fused at its N-terminus to EGFP were generated using the Custom Gene Synthesis Service from Integrated DNA Technologies (IDT). Twenty thousand cells were seeded in 24 well plates containing Poly-L-lysine Cellware 12 mm cover glasses (Corning). Twenty-four hours later, cells were transfected with 250 ng of plasmid using Lipofectamine (Thermo Fisher Scientific) in serum free media for 6 h. Then serum free media was replaced by supplemented DMEM and after 24 h of incubation, cells were either treated or not with 1 mM of the nitric oxide donor SIN-1 (Sigma-Aldrich) for another 24 h. Cells were then fixed with 4% paraformaldehyde for 15 min, incubated with 20 mM glycine for 15 min, were washed with PBS, permeabilized with PBS supplemented with 0.5% NP-40 (PBST) for 15 min and stained with a DAPI solution (1:1,000) in PBST for 10 minutes followed by a wash out step of 3 times with PBST. Finally, samples were mounted using antifade mounting medium Vectashield (VECTOR, #H-1000)) and stored in the dark at 4°C until imaging. Images were acquired using a STELLARIS 8 FALCON-STED (LEICA) confocal microscope in a 1,024 × 1,024-pixel format with Zoom 2 and an objective HC PL APOCS2 63x/1.30 GLYC. The pinhole value was 1, corresponding to 102.87 μm. The mean fluorescence intensity of the nuclear and cytosolic compartments was measured using ImageJ software. The green (Gapdh) and blue (DAPI) channels were used to create ROIs for the cytoplasmic and nuclear compartments, respectively. All images were subjected to Lightning Adaptive Deconvolution with the default settings of the Leica software.

### Statistical analysis

Depending on the number of independent variables, either one-way or two-way ANOVA was applied, followed by a Tukey multiple range test to determine differences between groups with Gaussian data distribution (square root transformation were employed for percentage data). The differences between 2 samples were analyzed by the Student’s *t* test. The contingency graphs were analyzed by the chi-squared (and Fisher exact) test.

## Supplementary Material

Supplementary Files

This is a list of supplementary files associated with this preprint. Click to download.

- SourcedataFigs110.xlsx
- TableS2.docx
- SourcedataFigsS1S16.xlsx
- FigurasS1S16GAPDHneutrophilR1.pdf
- Video1.mp4
- Video3.mp4
- Video5.mp4
- Video4.mp4
- Video2.mp4
- TableS1R1.docx
- rs.pdf

## Figures and Tables

**Figure 1.**
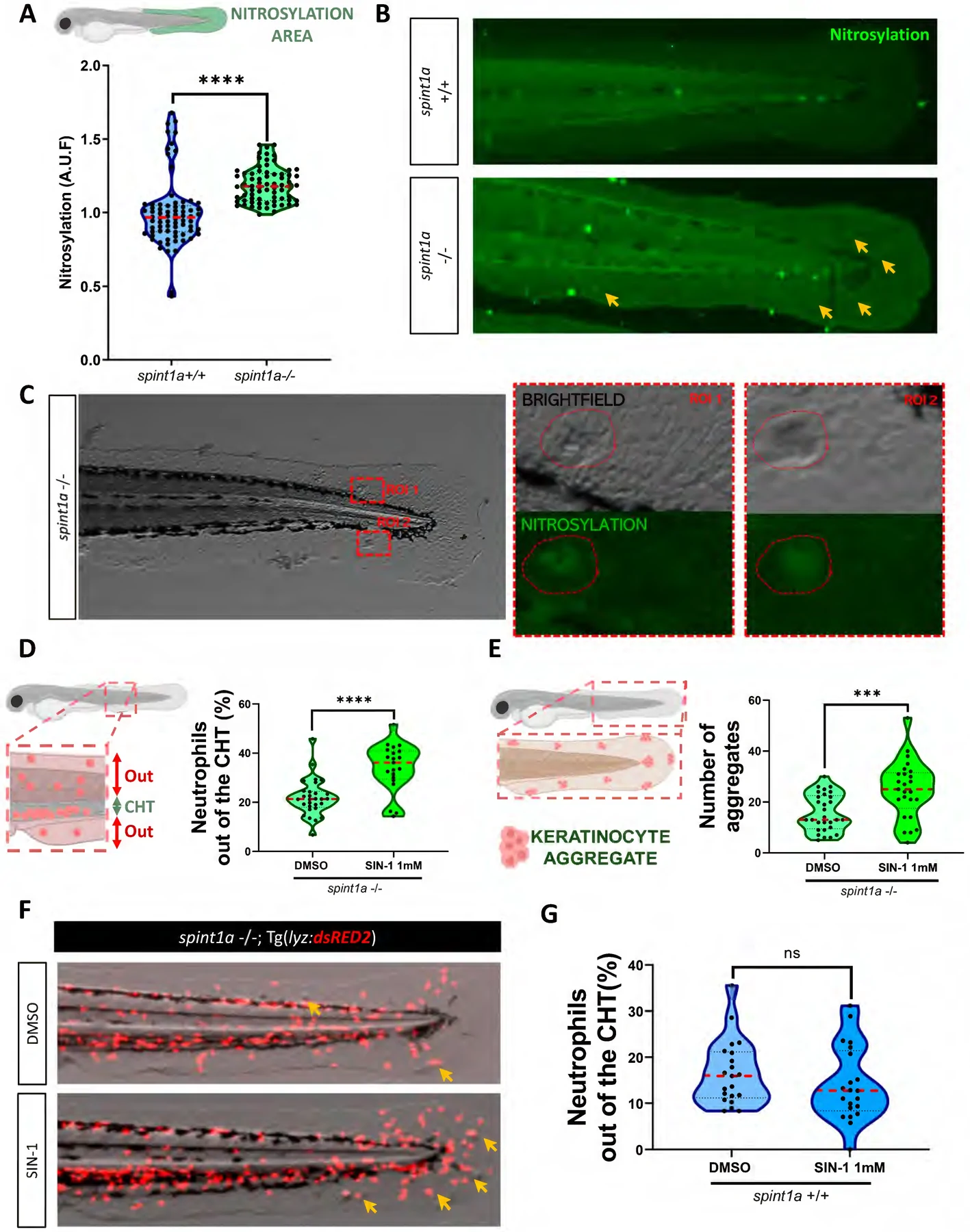
Nitrosative stress contributes to skin inflammation of Spint1a-deficient larvae. **(A-C)** Immunohistochemical analysis of nitrosylation in zebrafish *spint1a* +/+ and *spint1a* −/− larvae. **(A)** Nitrosylation levels in the tail measured by fluorescence intensity (*spint1a* +/+: n = 75, *spint1a* −/−: n = 71). **(B)** Representative images of nitrosylation in larval tail. Yellow arrows indicate the keratinocyte aggregates. (**C**) Detail at high magnification of 2 keratinocyte aggregates of a Spint1a-deficient larva brightly stained with the anti-nitrosylation antibody. **(D-F)** Zebrafish embryos of *spint1a* −/− line were treated for 48 h with 1 mM SIN-1. **(D)** Percentage of neutrophils out of the CHT (DMSO: n = 31, SIN-1: n = 21) and **(E)** number of keratinocyte aggregates (arrows) in the tail (DMSO: n = 29, SIN-1: n = 25). **(F)** Representative images showing neutrophils and the skin of Spint1a-deficient larvae treated with DMSO and SIN-1. **(G)** Percentage of neutrophils infiltrated in the skin of control *spint1a* +/+ larvae treated with SIN-1 (DMSO: n = 22, SIN-1: n = 22). Each dot represents one individual, and the median for each group is also shown. p-Values were calculated using Student’s *t* test. Ns, non-significant; ***p ≤ 0.001; ****p ≤ 0.0001. A.U.F., Arbitrary Units of Fluorescence. DMSO, dimethyl sulfoxide; CHT, caudal hematopoietic tissue; SIN-1, linsidomine chlorhydrate.

**Figure 2.**
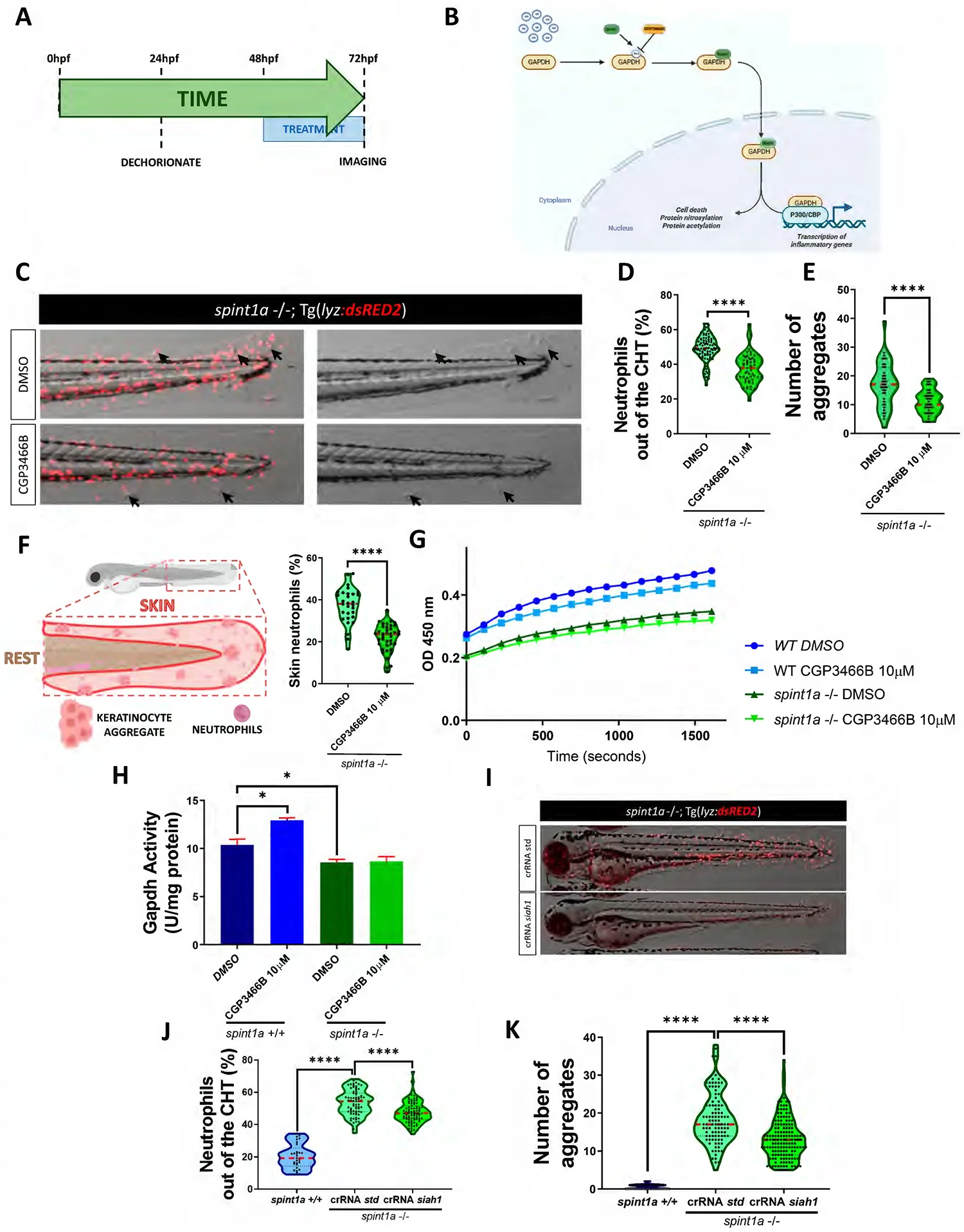
Inhibition of Gapdh nuclear translocation alleviates skin inflammation. **(A-G)** Zebrafish embryos were dechorionated at 24 hpf and treated with the inhibitor CGP3466B from 48 to 72 hpf. **(B)** CGP3466B inhibits GAPDH nitrosylation in the Cys^152^ residue of the protein, impairing its SIAH1-dependent nuclear translocation. **(C-D)** Representative merge images and quantitation of neutrophil dispersion (DMSO: n = 89, CGP3466B: n = 52) **(D)**, keratinocyte aggregates number (DMSO: n = 39, CGP3466B: n = 39) **(E)** and skin infiltrated neutrophils (DMSO: n = 30, CGP3466B: n = 39) (**F**) of Spint1a-deficient larvae treated with either DMSO or 10 μM CGP3466B. Black arrows indicate keratinocyte aggregates. **(G, H)** Gapdh enzymatic activity assay showing optical density at 450 nm with time (N = 2) **(G)** and quantitation of Gapdh enzymatic activity (N = 2) **(H)** in wild type and Spint1a-deficient larvae treated with either DMSO or CGP3466B. **(I-K)** Representative merge images showing neutrophils and the skin of Spint1a-deficient larvae microinjected with a standard (std) or *siah1* crRNA/Cas9 complexes **(I)**. Percentage of dispersed neutrophils (*spint1a*+/+: n = 29, *spint1a*−/− + crRNA *std:* n = 72, *spint1a*−/− + crRNA *siah1:* n = 86) **(J)** and number of keratinocyte aggregates (*spint1a*+/+: n = 64, crRNA *std*: n = 100, crRNA *siah1*: n = 153) **(K)** of wild type and Siah1-deficient larvae. Each dot represents one individual, and the median (D, E) or mean ± SD (H) for each group is also shown. p-Values were calculated using ANOVA and Tukey multiple range test. *p≤0.05; ****p ≤ 0.0001. DMSO, dimethyl sulfoxide; crRNA, CRISPR RNA; OD, Optical Density; U, Unit of enzymatic activity.

**Figure 3.**
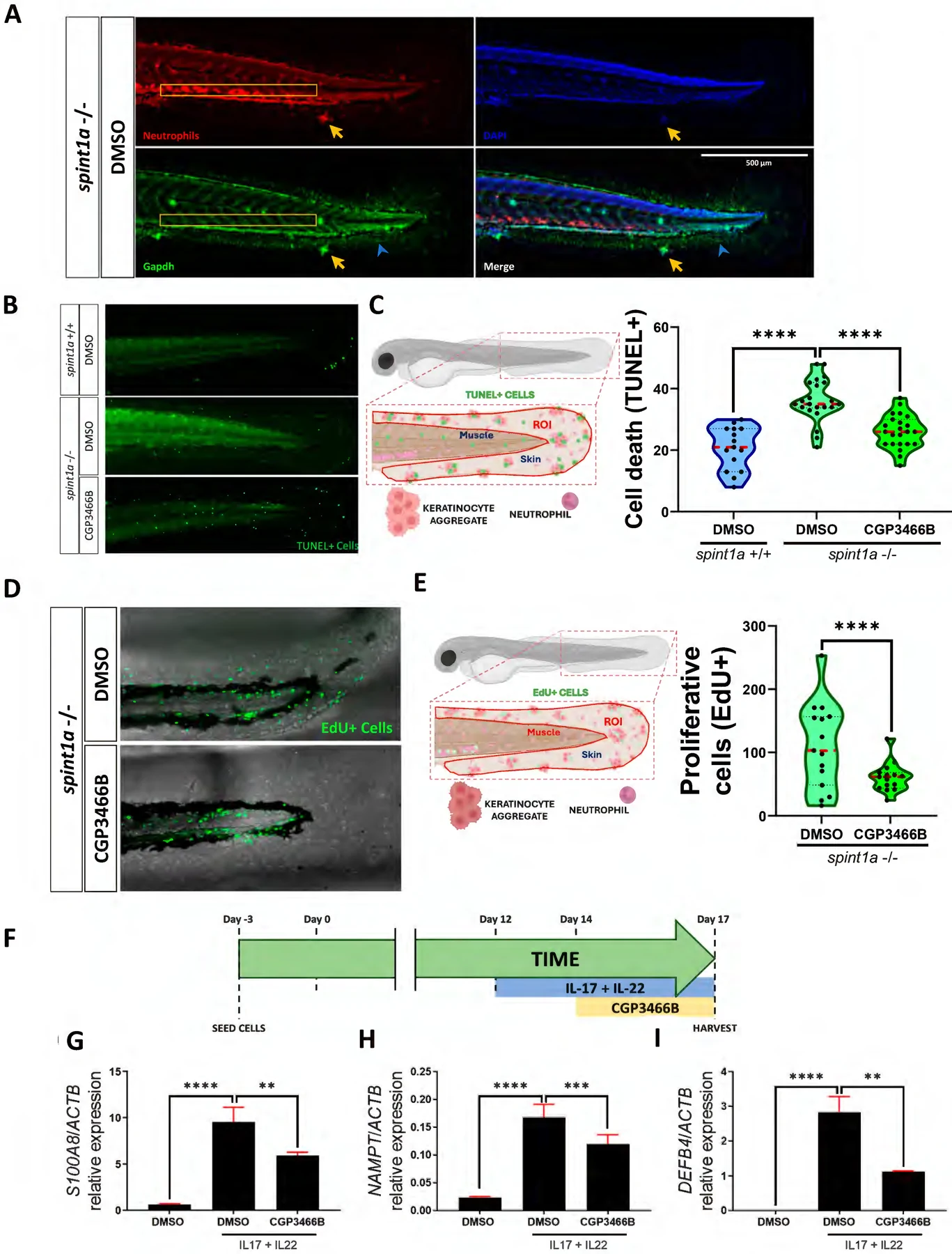
Inhibition of Gapdh nuclear translocation alleviates skin inflammation in zebrafish and human. **(A-E) (A)** Representative images of Gapdh immunolocalization of spint1a −/− larval tails at 72 hpf. Arrow labels a keratinocyte aggregate with several neutrophils showing robust nuclear Gapdh staining. In contrast, neutrophils located at the CHT (boxed) were hardly stained. Arrowhead labels skin keratinocytes showing heterogeneous staining. **(B)** Representative images of TUNEL assay of *spint1a* +/+ and *spint1a* −/− larval tails at 72 hpf treated with DMSO and 10 μM CGP3466B. **(C)** Quantitation of the number of dead cells (TUNEL^+^) observed in the tail skin (ROI) of Spint1a-deficient and wild type larvae (*spint1a* +/+: n = 16, *spint1a* −/− DMSO: n = 24, *spint1a* −/− CGP3466B: n = 24). **(D)** Representative images of EdU^+^ cells of *spint1a* −/− larvae treated with either DMSO or 10 μM CGP3466B. **(E)** Quantitation of the number of proliferating cells (EdU^+^) observed at 72 hpf on the tail skin (ROI) (DMSO: n = 15, CGP3466B; n = 15). Each dot represents one individual, and the median for each group is also shown. p-Values were calculated using Student’s *t* test or ANOVA and Tukey multiple range test. ****p ≤ 0.0001. DMSO, dimethyl sulfoxide; TUNEL, Terminal deoxynucleotidyl transferase dUTP nick-end labeling; EdU, 5-ethynyl-20- deoxyuridine. **(F-I)** After seeding, human keratinocytes were growth for 3 d, differentiated by air exposure for 12 d and then stimulated with IL-17 and IL-22 to induce psoriasis-like inflammation **(F)**. At day 14, after 2 d with the inflammatory stimuli, keratinocytes were treated with 100 μM CGP3466B until day 17 when they were harvested and processed for RNA extraction. The transcript levels of the genes *S100A8* (N = 3) **(G)**, *NAMPT* (N = 3) **(H)**, and *DEFB4* (N = 3) **(I)** were analyzed by RT-qPCR. The median (D, E, J, K) and mean ± SD (H) for each group is shown. p-Values were calculated using Student’s *t* test (E) and ANOVA and Tukey multiple range test (C, G-I). **p ≤ 0.01, ***p ≤ 0.001, ****p ≤ 0.0001. DMSO, dimethyl sulfoxide; ROI, region of interest.

**Figure 4.**
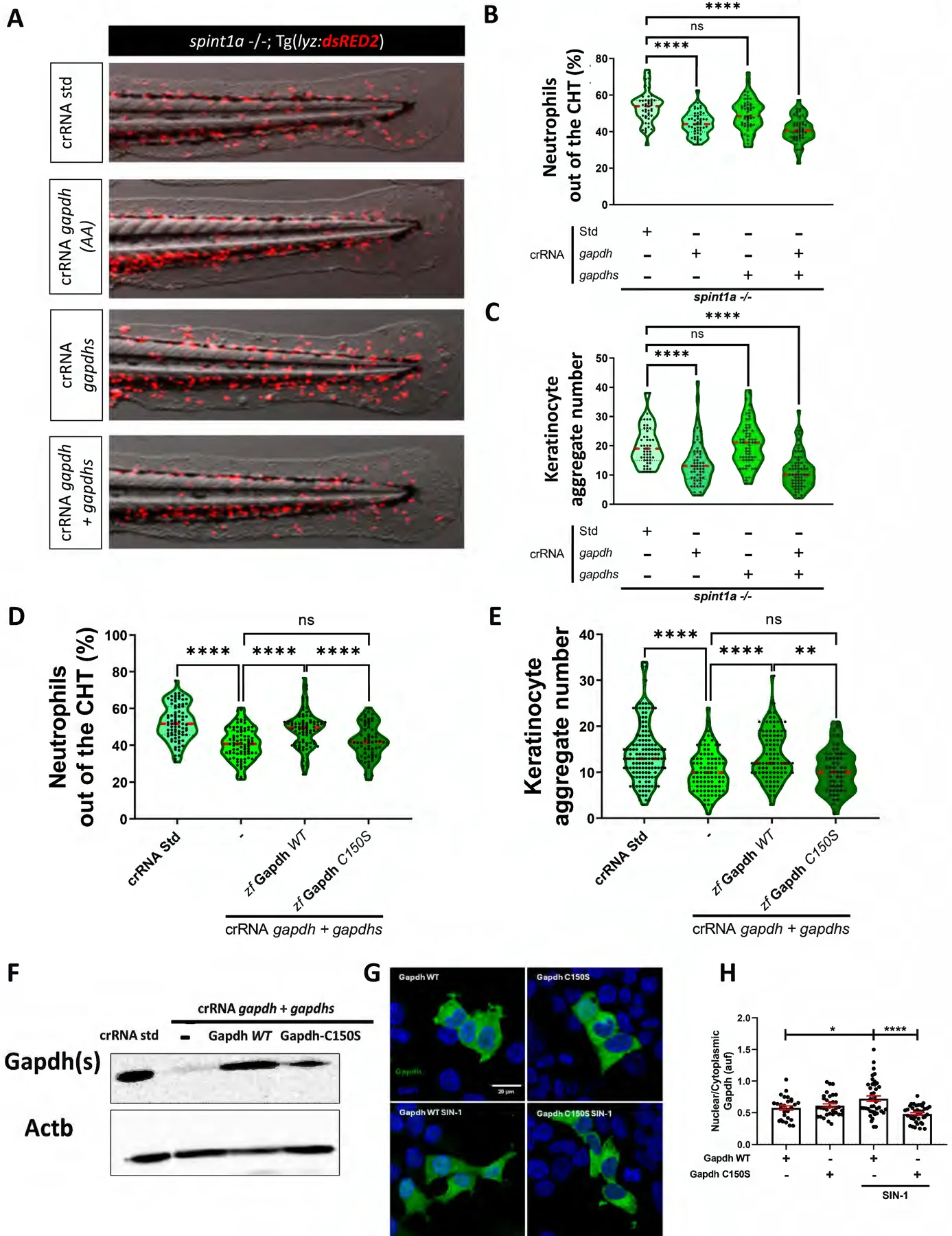
Wild type Gapdh, but not nuclear translocation defective Gapdh-C150S, reverses the anti-inflammatory effect of Gapdh/Gapdhs deficiency. **(A-C)** Representative merge images showing the skin and neutrophils of 3 dpf Spint1a-deficient larvae microinjected with either standard or *gapdh* and/or *gapdhs* crRNAs/Cas9 complexes. **(B)** Quantitation of neutrophil dispersion (crRNA *std*: n = 47, crRNA *gapdh*: n = 49, crRNA *gapdhs:* n = 51, crRNA *gapdh* + *gapdhs*: n = 50) **(B, D)** and number of keratinocyte aggregates in the skin (crRNA *std*: n = 49, crRNA *gapdh*: n = 57, crRNA *gapdhs*: n = 59, crRNA *gapdh* + *gapdhs*: n = 61) **(C)**. **(D-F)** Gapdh/Gapdhs-deficient larvae were forced to express mRNAs encoding either wild type Gapdh or Gapdh-C150S and neutrophil dispersion (crRNA *Std*: n = 81, crRNA *gapdh* + *gapdhs*: n = 89, crRNA *gapdh* + *gapdhs* + *gapdh* WT: n = 84, crRNA *gapdh* + *gapdhs* + *gapdh* C150S: n = 61) **(D)**, the number of keratinocyte aggregates (crRNA *Std*: n = 115, crRNA *gapdh* + *gapdhs*: n = 93, crRNA *gapdh* + *gapdhs* + *gapdh* WT: n = 99, crRNA *gapdh* + *gapdhs* + *gapdh* C150S: n = 53) **(E)** and Gapdh and Gapdhs protein amounts **(F)** were analyzed at 3 dpf. **(G, H)** HEK293T cells were transfected with zebrafish wild-type or C150S Gapdh fused to GFP and, after 24 h, treated with SIN-1 for an additional 24 h. Nuclear-to-cytoplasmic Gapdh ratios were then analyzed by confocal microscopy after counterstained the nuclei with DAPI (Gapdh: n = 27, Gapdh-C150S: n = 33, Gadph + SIN-1: n=42, Gapdh-C150S + SIN-1: n = 38). Each dot represents a larva (B-E) or a cell (H) and the median (B-E) and mean ± SD (H) for each group is also shown. p-Values were calculated using ANOVA and Tukey multiple range test. ns, non-significant; **p ≤ 0.01; ****p ≤ 0.0001. crRNA, CRISPR RNA; CHT, caudal hematopoietic tissue; std, standard; Gapdh, glyceraldehyde-3-phosphate dehydrogenase; Gapdhs, glyceraldehyde-3-phosphate dehydrogenase, spermatogenic; Actb, β-actin.

**Figure 5.**
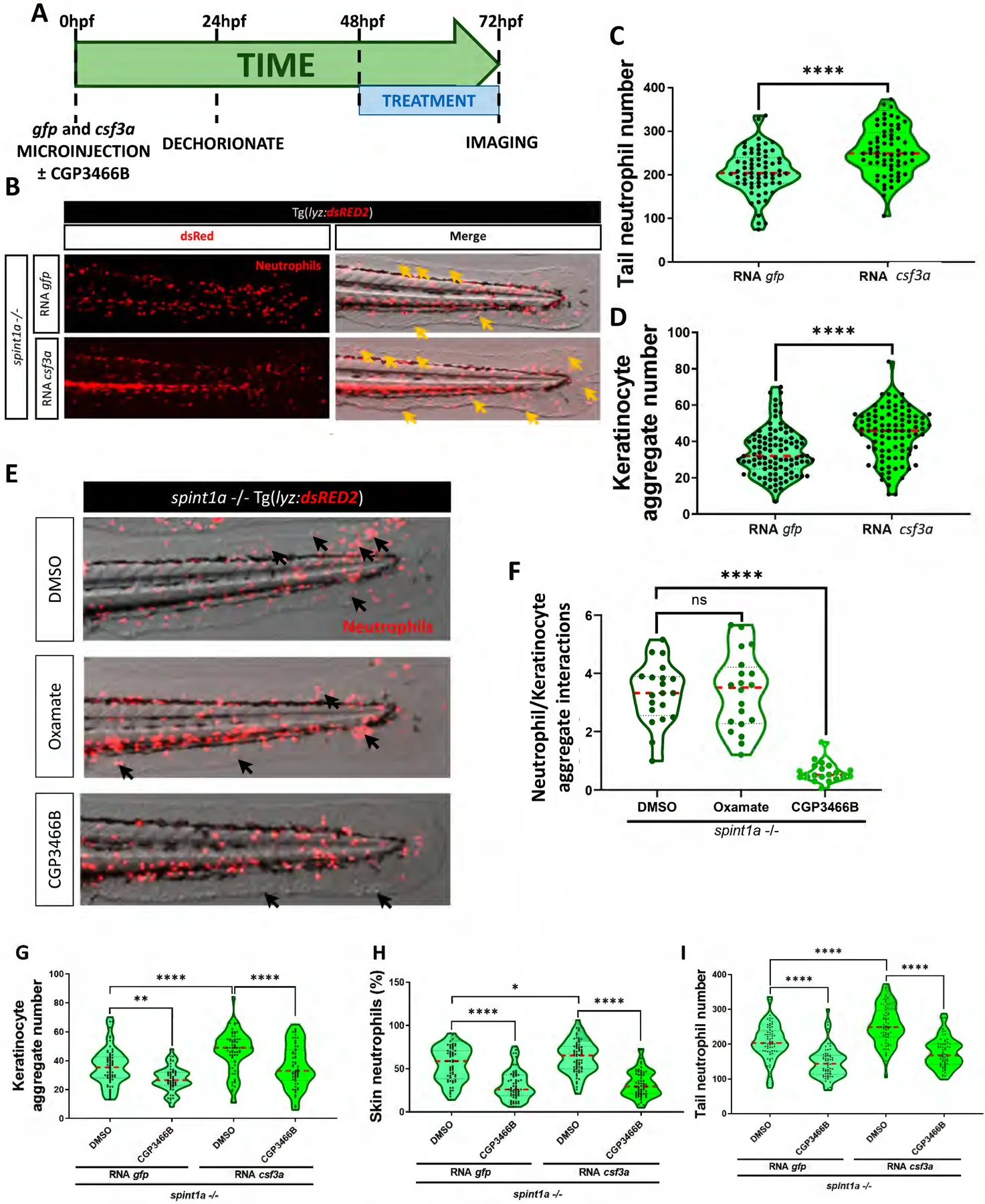
Pharmacological inhibition of Gapdh nuclear translocation impairs neutrophil skin infiltration and their interactions with keratinocyte aggregates in the inflamed skin. **(A)** Spint1a-deficient zebrafish embryos were microinjected in one-cell stage with *csf3a* and *gfp* mRNAs **(A-D, G-I)** or left uninjected **(E-F)**, dechorionated at 24 hpf, treated with DMSO, 10 μM oxamate or 10μM CGP3466B at 48 hpf, and imaged and analyzed at 72 hpf. **(B, E)** Representative merge images showing skin and neutrophils in Spint1a-deficient larvae treated with DMSO, oxamate or CGP3466B. Arrows indicate keratinocyte aggregates. **(C, I)** Total neutrophil number at the larval tail (RNA *gfp*: n = 65, RNA *csf3a*: n = 64). **(H)** Percentage of neutrophils recruited to the skin (RNA *gfp*: n = 99, RNA *csf3a*: n = 101). **(F)** Neutrophil/keratinocyte aggregate interactions (DMSO: n = 20, Oxamate: n = 20, CGP3466B: n = 20). **(D, G)** Keratinocyte aggregates (RNA *gfp* DMSO: n = 68, RNA *gfp* CGP3466B: n = 54, RNA *csf3a* DMSO: n = 64, RNA *csf3a* CGP3466B: n = 60). **(I)** Total neutrophils at the larval tails (RNA *gfp* DMSO: n = 61, RNA *gfp* CGP3466B: n = 59, RNA *csf3a* DMSO: n = 64, RNA *csf3a* CGP3466B: n = 60). Each dot represents one individual, and the median for each group is also shown. p-Values were calculated using Student’s *t* test (C, D) and ANOVA followed by Tukey multiple range test (G-I). ns, non-significant; *p ≤ 0.05; **p ≤ 0.01; ****p ≤ 0.0001. DMSO, dimethyl sulfoxide; CHT, caudal hematopoietic tissue.

**Figure 6.**
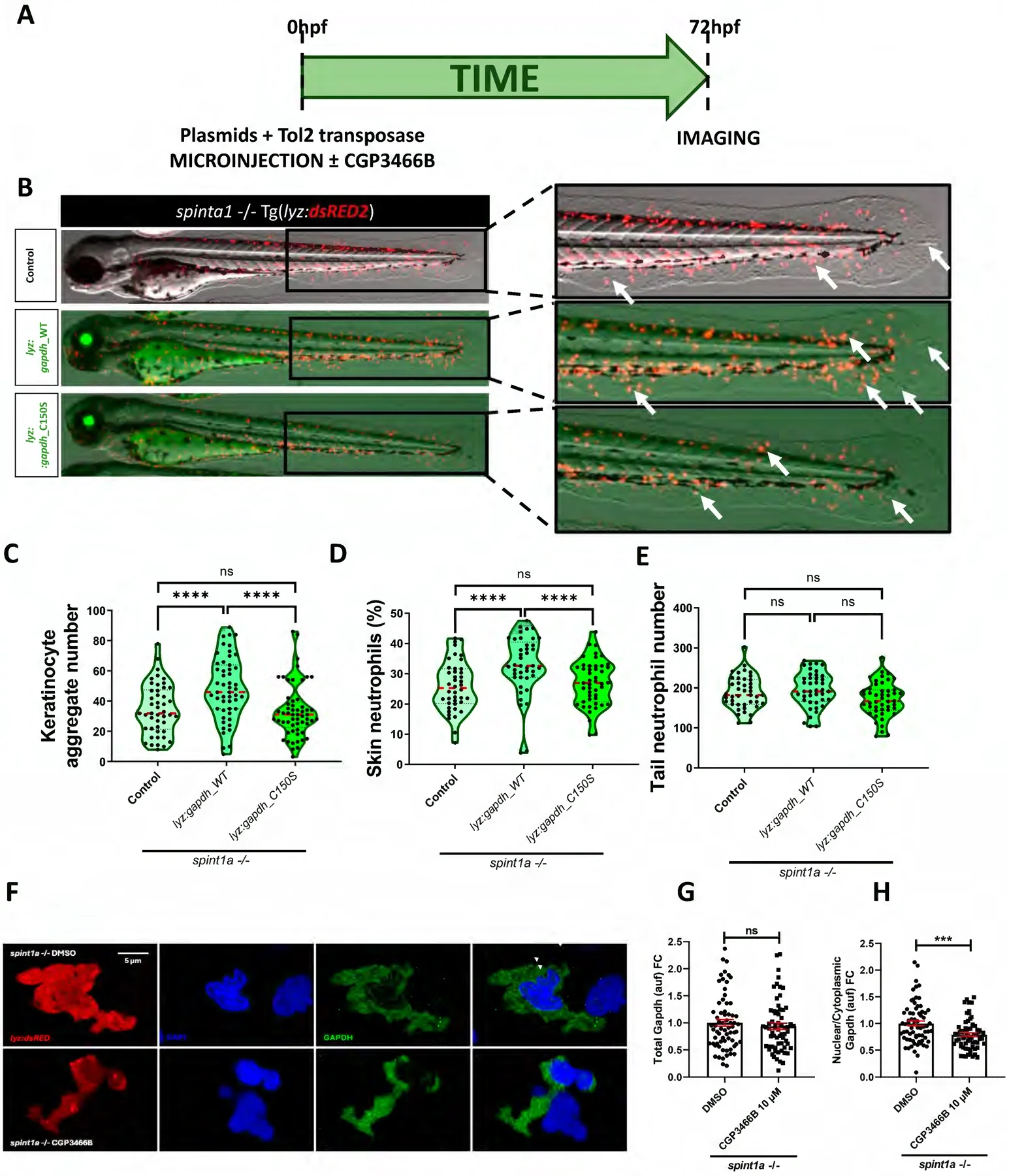
Gapdh nuclear translocation regulates in a cell-autonomous manner skin neutrophil infiltration. **(A-E)** Spint1a-deficient embryos were microinjected with plasmids driven either wild type or C150S Gapdh driven by the *lysozyme* promoter and containing the *cry:ECFP* reporter for screening together with Tol2 transposase RNA. **(B)** Representative merged images showing skin, neutrophils, and labeled crystallin of 3 dpf larvae. White arrows label the keratinocyte aggregates in the skin. (C) Number of keratinocyte aggregates (Control: n = 46, *lyz:gapdh*_WT: n = 51, *lyz:gapdh*_C150S: n = 57). **(D)** Percentage of neutrophils in the skin (Control: n = 42, *lyz:gapdh*_WT: n = 41, *lyz:gapdh*_C150S: n = 47). **(E)** Total number of neutrophils (Control: n = 42, *lyz:gapdh*_WT: n = 41, *lyz:gapdh*_C150S: n = 47). **(F-H)** Spint1a-deficient; *Tg(lyz:dsRED)* larvae were treated with either DMSO or CGP3466B from 48 to 72 hpf and then immunostained with an anti-Gapdh antibody. Nuclei were counterstained with DAPI. Note the nuclear Gapdh was localized in euchromatine (arrowheads). About 4–5 neutrophils were analyzed per larva, and 4–5 larvae per group in each experiment. Each dot represents one individual (C-E) or a neutrophil (G, H), and the median (C-E) and median ± SD (G, H) for each group is also shown. p-Values were calculated using ANOVA followed by Tukey multiple range test (C-E) or Student’s *t* test (G, H). ns, non-significant; **p ≤ 0.01; ***p ≤ 0.001 ****p ≤ 0.0001. *lyz, lysozyme;* WT, wild type*; rps11, ribosomal protein S11; gapdh, glyceraldehyde 3-phosphate dehydrogenase*; FC, fold change.

**Figure 7.**
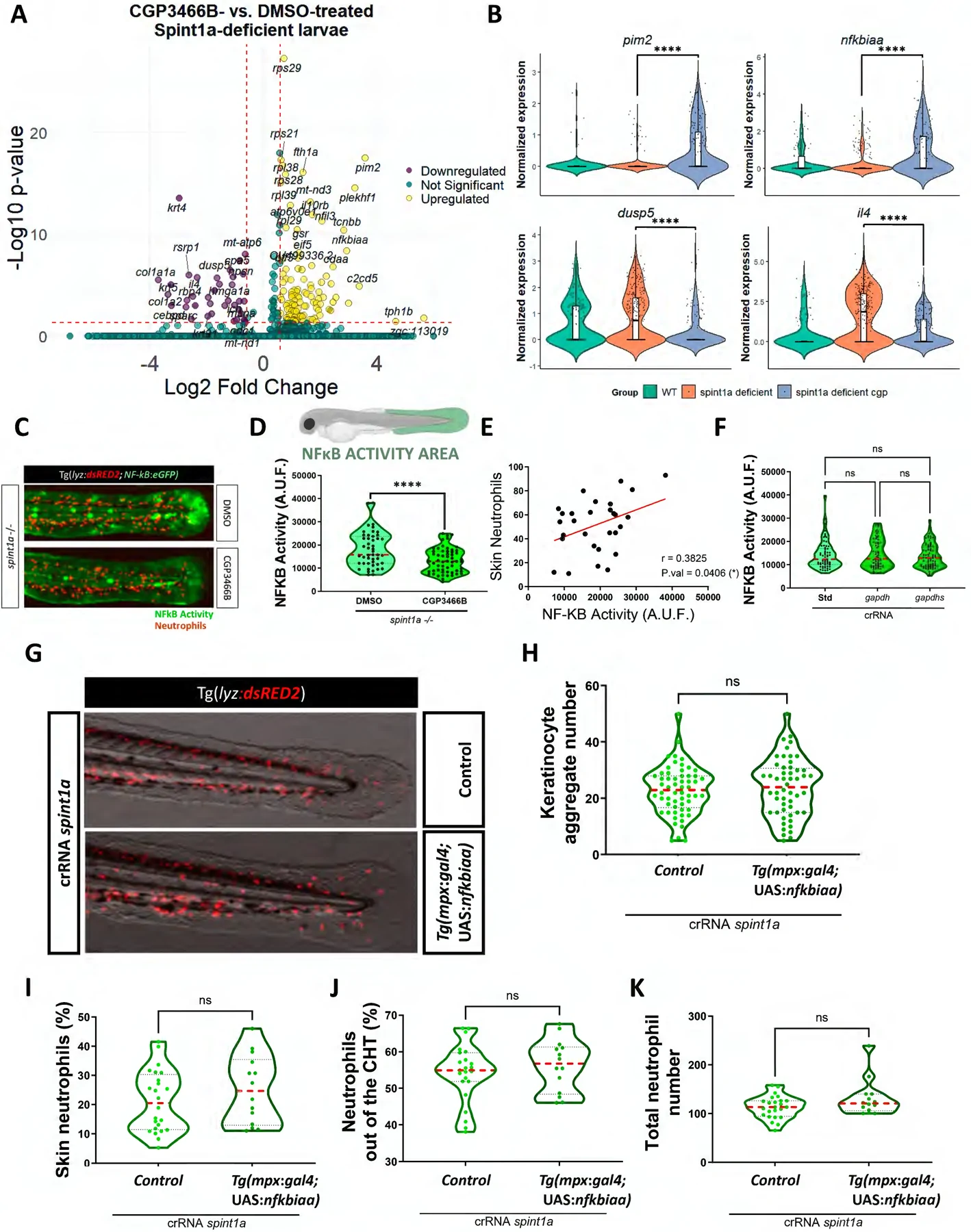
NF-κB is not involved in the regulation of the proinflammatory activity of neutrophils by nuclear translocation of Gapdh. **(A, B) (A)** Volcano plot resulting from the single cell RNA-seq analysis of neutrophils treated with either DMSO or 10 μM CGP3466B Spint1a-deficient. Upregulated (orange) and downregulated (purple) genes in neutrophils from Spint1a-deficient larvae treated with CGP3466B compared to controls. **(B)** Violin Plot representing several DEGs in neutrophils from wild type (WT) and Spint1a-defcient larvae treated with either DMSO or CGP3466B. The genes *pim2* and *nfkbiaa* were upregulated after the treatment with CGP3466B, while the genes *dusp5* and *il4* were downregulated. Each dot represents the gene expression level in a different cell analyzed. p-Values were calculated using Wilcoxon Rank Sum test. **(C, D)** Representative merge images of NF-κB activity and neutrophils **(C)** and violin plot showing NF-κB activity fluorescence intensity (DMSO: n = 47, CGP3466B: n = 57) **(D)** of 3 dpf Spint1a-deficient larvae treated either with DMSO or 10 μM CGP3466B. **(E)** Correlative variation between the number of skin neutrophils and NF-κB activity in untreated Spint1a-deficient larvae (n = 47). **(F)** Violin plot showing NF-κB activity in the skin of Spint1a-deficient larvae after genetic inhibition of *gapdh* and *gapdhs* by injection one-cell stage embryos with control (std), *gapdh* or *gapdhs* crRNA/Cas9 complexes (Std: n = 62, *gapdh*: n = 62, *gapdhs*: n = 72). **(G-K)** Representative merge images **(G)** and violin plot showing the number of keratinocyte aggregates (Control: n = 58, *mpx:gal4 x UAS:DN-ikbaa*: n = 52) **(H)**, the percentage of skin neutrophils (Control: n = 24, *mpx:gal4 x UAS:DN-ikbaa*: n = 14) **(I)**, the percentage of dispersed neutrophils (Control: n = 24, *mpx:gal4 x UAS:DN-ikbaa*: n = 14)**(J)** and the total number of neutrophils (Control: n = 24, *mpx:gal4 x UAS:DN-ikbaa*: n = 14) **(K)** of 3 dpf wild type (control) and *Tg(mpx:gal4;UAS:nfkbiaa)* larvae upon genetic inhibition of *spint1a* by injection one-cell stage embryos with crRNA/Cas9 complexes. Each dot represents one individual, and the median for each group is also shown. p-Values were calculated using Student’s *t* test (B, D, H-K), Pearson correlation (E) or ANOVA followed by Tukey multiple range test (F). ns, non-significant; * p ≤ 0.05; ****p ≤ 0.0001. p-Values were calculated using t test. crRNA; CRISPR RNA; CHT, Caudal Hematopoietic Tissue; WT, wild type. DMSO, dimethyl sulfoxide; *gapdh*, glyceraldehyde 3-phosphate dehydrogenase; *gapdhs*, glyceraldehyde 3-phosphate dehydrogenase, spermatogenic; A.U.F., Arbitrary Units of Fluorescence.

**Figure 8.**
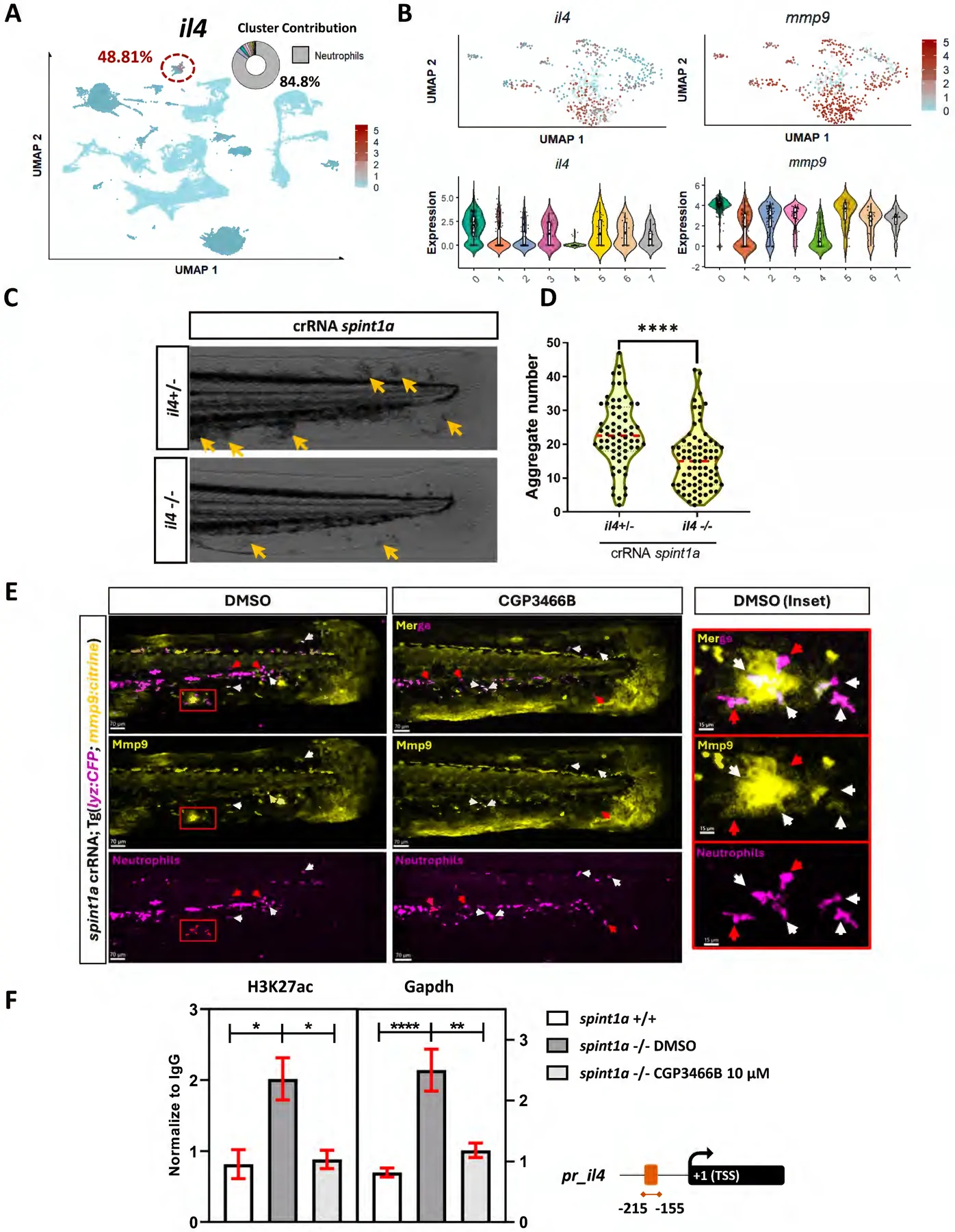
Nuclear translocation of Gapdh induces *Il4* gene expression in neutrophils contributing to skin inflammation. **(A, B)** Density plot showing *il4* in all cell clusters **(A)** and *mmp9* and *il4* in neutrophils **(B)** of the scRNA-seq analysis. Each dot represents the gene expression level in a different cell analyzed. **(C, D)** Representative images **(C)** and violin plot representing the number of keratinocyte aggregates (*il4a* +/−: n = 66, *il4a* −/−: 77) **(D)** of *il4* +/− and *il4* −/− larvae upon genetic inhibition of *spint1a* by injecting one-cell stage embryos with crRNA/Cas9 complexes. Yellow arrows indicate the keratinocyte aggregates in the skin. Each dot represents one individual, and the median for each group is also shown. p-Values were calculated using Student’s *t* test. ****p ≤ 0.0001. crRNA, CRISPR RNA; *il4*, interleukin 4. **(E)** Representative merged images showing neutrophils (magenta) and *mmp9* (yellow) in 3 dpf Spint1a-deficient larvae treated with DMSO or CGP3466B for 24 h. Red arrows indicate *mmp9*^−^ neutrophils and white arrows indicate *mmp9*^+^ neutrophils. Higher magnification of the keratinocyte aggregate boxed in the DMSO-treated Spint1a-deficient larva is also shown in the right panel. **(F)** Analysis of acetylation of histone H3K27 (H3K27ac) and Gapdh recruitment to *il4* promoter region of 3 dpf wild type and Spint1a-deficient larvae treated with DMSO or CGP3466B for 24 h. The assessed *il4* promoter region is also shown. The orange box indicates the binding site located between position −215 and −155 of the transcription start site (TSS). The mean ± SD for each group is shown of 2 independent CUT&RUN assay performed in triplicate. p-Values were calculated using ANOVA and Tukey multiple range test. *p ≤ 0.05, **p ≤ 0.01, ****p ≤ 0.0001.

**Figure 9:**
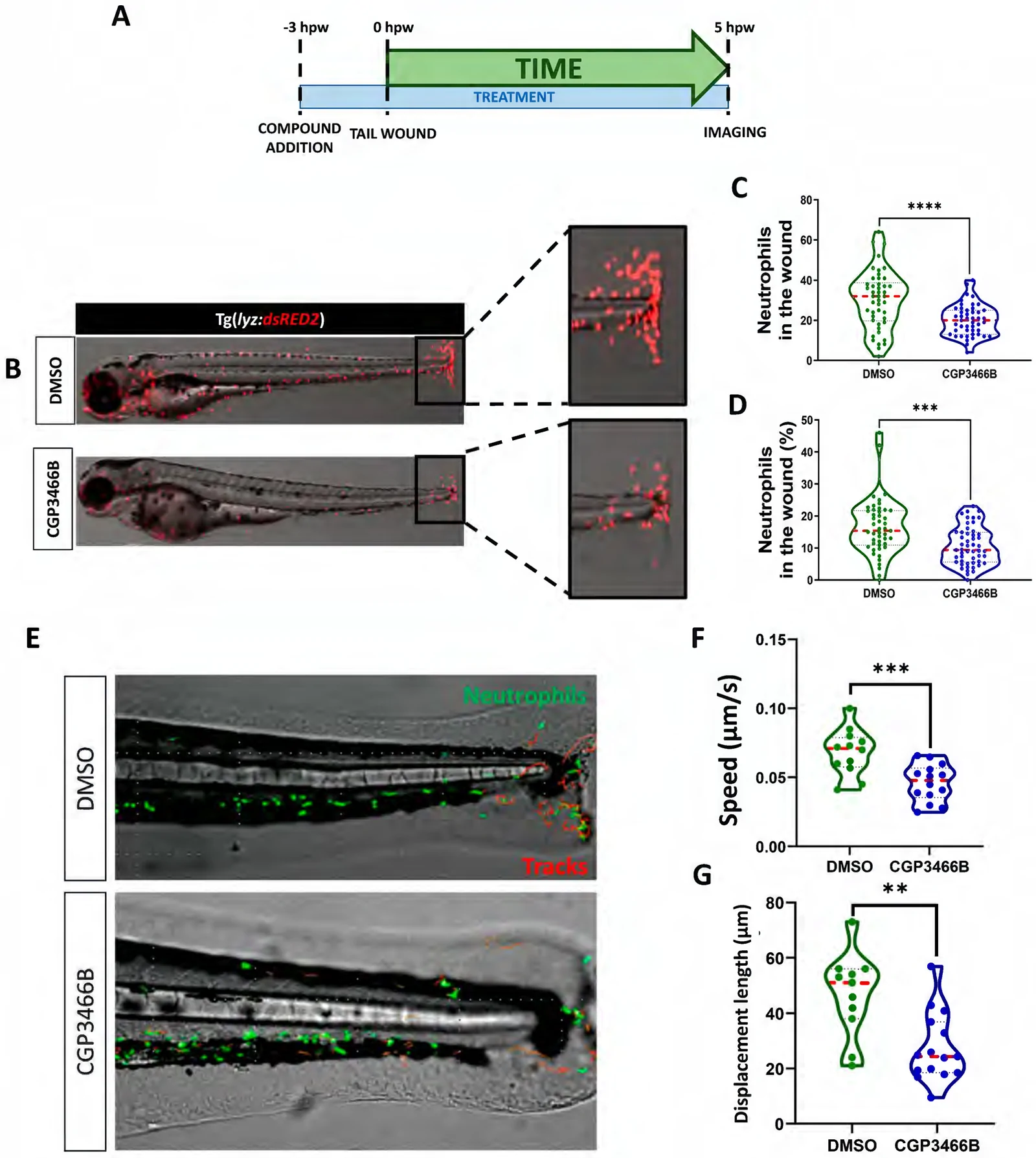
Pharmacological inhibition of Gapdh nuclear translocation impairs neutrophil recruitment to wound. **(A-D)** Three dpf larvae pre-treated for 3 h with 10 μM CGP3466B were tail wounded, imaged and the neutrophils recruited were quantified at 5 hpw **(A)**. Representative merge images **(B)**, total number of neutrophils recruited to the wound (DMSO: n = 46, CGP3466B: n = 47) **(C)** and percentage of recruited neutrophils at 5 hpw **(D)** of DMSO (control) and CGP3466B-treated larvae (DMSO: n = 52, CGP3466B: n = 47). **(E-G)** Representative merge images showing the tails, neutrophils, and their respective tracks in dragon tail format of DMSO- and CGP3466B-treated larvae at 5 hpw **(E)**. The mean speed of the movement of all the neutrophils (DMSO: n = 12, CGP3466B: n = 14) **(F)** and the length of the displacement **(G)** measured by the analysis of their tracks (DMSO: n = 11, CGP3466B: n = 15). Each dot represents one individual, and the median for each group is also shown. p-Values were calculated using Student’s *t* test. **p ≤ 0.01, ***p ≤ 0.001, ****p ≤ 0.0001. hpw, hours post-wounding; DMSO, Dimethyl sulfoxide.

**Figure 10.**
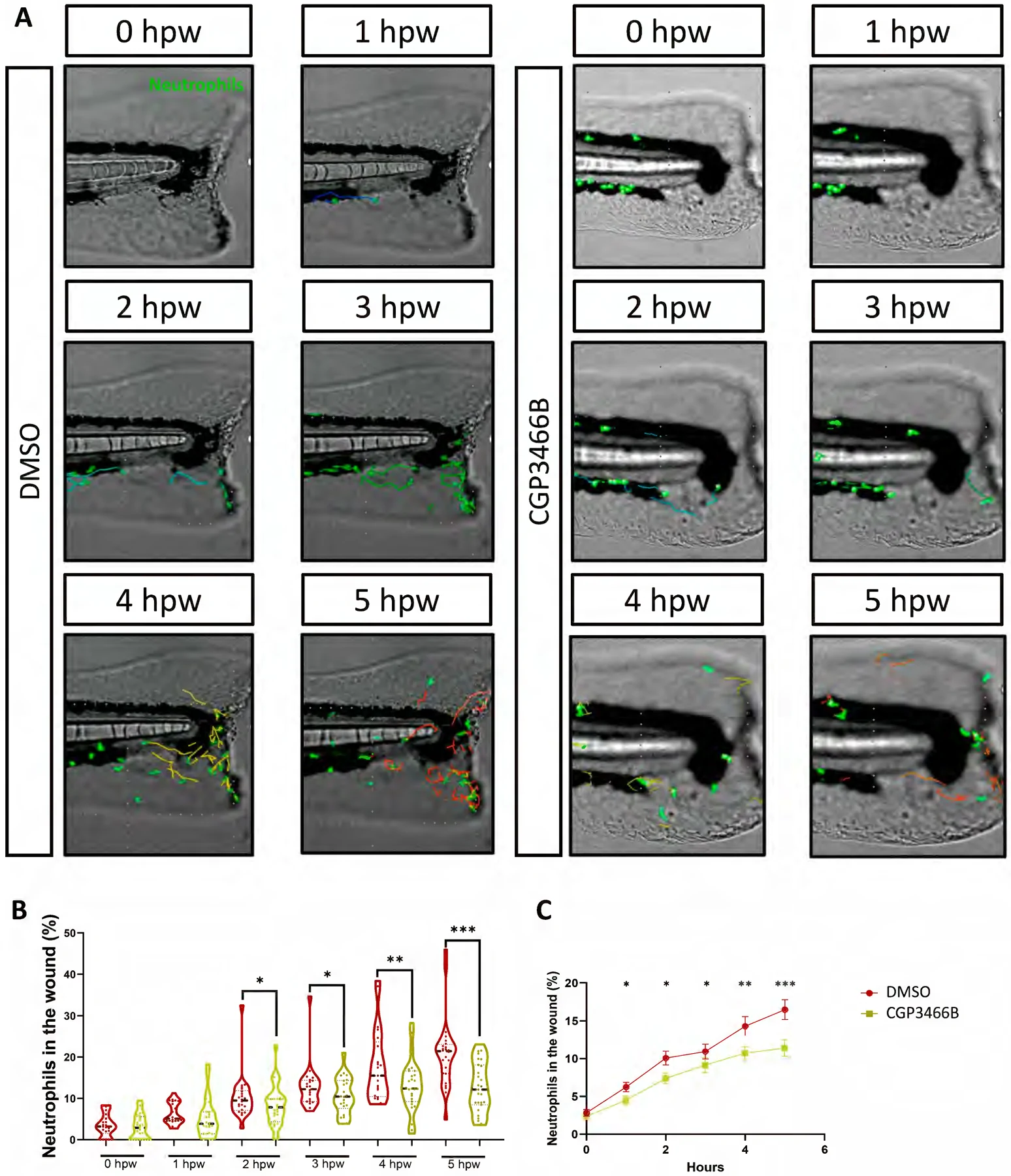
Neutrophil recruitment dynamics to wound are altered after the treatment with CGP3466B. **(A)** Representative merge images showing the tails, the neutrophils and their tracks in dragon tail format of DMSO- and CGP3466B-treated larvae at 0, 1, 2, 3, 4 and 5 hpw. **(B, C)** The percentage of neutrophils recruited to the tail was measured at each time point and condition (DMSO: n = 24, CGP3466B: n = 30). Each dot represents one individual, and the median for each group is also shown. p-Values were calculated using Student’s *t* test comparing the two samples at the same time point. *p ≤ 0.05, **p ≤ 0.01, ***p ≤ 0.001. hpw, hours post-wounding; DMSO, dimethyl sulfoxide.

## Data Availability

Source data are provided with this paper.
